# A chromosome-level genome assembly reveals genomic characteristics of the American mink (*Neogale vison*)

**DOI:** 10.1038/s42003-022-04341-5

**Published:** 2022-12-16

**Authors:** Karim Karimi, Duy Ngoc Do, Jingy Wang, John Easley, Shima Borzouie, Mehdi Sargolzaei, Graham Plastow, Zhiquan Wang, Younes Miar

**Affiliations:** 1 Department of Animal Science and Aquaculture, Dalhousie University, Truro, NS Canada; 2 Joint Mink Research Committee, Fur Commission USA, Preston, ID USA; 3 Mink Veterinary Consulting and Research Service, Plymouth, WI USA; 4 Department of Pathobiology, University of Guelph, Guelph, ON Canada; 5 Select Sires Inc., Plain City, OH USA; 6 Livestock Gentec, Department of Agricultural, Food and Nutritional Science, University of Alberta, Edmonton, AB Canada

**Keywords:** Genome informatics, Genetics

## Abstract

Availability of a contiguous chromosome-level genome assembly is the foundational step to develop genome-based studies in American mink (*Neogale vison*). The main objective of this study was to provide a high quality chromosome-level genome assembly for American mink. An initial draft of the genome assembly was generated using 2,884,047 PacBio long reads. Integration of Hi-C data into the initial draft led to an assembly with 183 scaffolds and scaffold N50 of 220 Mb. This gap-free genome assembly of American mink (ASM_NN_V1) had a length of 2.68 Gb in which about 98.6% of the whole genome was covered by 15 chromosomes. In total, 25,377 genes were predicted across the American mink genome using the NCBI Eukaryotic Genome Annotation Pipeline. In addition, gene orthology, demographic history, synteny blocks, and phylogenetic relationships were studied in connection with the genomes of other related *Carnivora*. Furthermore, population-based statistics of 100 sequenced mink were presented using the newly assembled genome. Remarkable improvements were observed in genome contiguity, the number of scaffolds, and annotation compared to the first draft of mink genome assembly (NNQGG.v01). This high-quality genome assembly will support the development of efficient breeding strategies as well as conservation programs for American mink.

## Abstract (web summary)

A chromosome-level genome assembly of the American mink (*Neogale vison*) provides a valuable genetic resource to guide mink breeding strategies and conservation efforts.

## Introduction

American mink (*Neogale vison*) is a carnivorous mammal native to North America and is a member of the family *Mustelidae*^1^. American mink has been raised in captivity since the middle 19th century owing to having one of the most desirable sources of fur in the world^2^. The increasing cost of fur production and emerging diseases (e.g., Aleutian disease and coronavirus) are the most important challenges affecting the mink industry in recent years^3^. Although the mink industry requires an efficient breeding program to deal with the aforementioned issues^4^, modern breeding strategies have not been broadly developed in this industry. For instance, whereas genomic selection is being used as the routine strategy to select superior animals in livestock species e.g., dairy cattle^5^, pig^6^, and sheep^7^, this approach has not been widely applied in the mink industry. Knowledge of the genome would facilitate efforts to improve the economically important traits and design conservation programs for American mink. Availability of high-quality genome assembly is not only essential to develop genomic research for American mink but also would be a valuable resource for comparative genomics and evolutionary studies in carnivorous mammals. Furthermore, American mink could be used as an animal model to study diseases shared by human and mink e.g., deafness^8^, Chediak-Higashi syndrome^9^, and coronavirus susceptibility^10,11^.

Advances in DNA sequencing technologies provided the opportunity to sequence complex genomes, driving a wide range of biological discoveries. The fast development of third-generation sequencing has overcome the initial limitations in both accuracy and length of long reads, making it possible to provide reliable genome assembly for different species. The long reads resolve the reconstruction problem of the repetitive genomic regions, leading to more contiguous genome structures and higher mapping certainty^12^. Currently, Pacific Biosciences (PacBio) and Oxford Nanopore are the most popular technologies to generate long reads. The PacBio Single- Molecule High-Fidelity (HiFi) reads provide base-level resolution with >99.9% read accuracy, enabling high-quality de novo genome assembly that would guarantee the accuracy of structural variants and transcript isoforms identified in the subsequent analyses^13^. However, even using the long reads, the genome contiguity can be interrupted by repetitive genomic regions e.g., centromeres and long interspersed nuclear elements (LINEs), which makes it necessary to use scaffolding technologies. High-throughput chromosome conformation capture (Hi-C) technology is a complementary approach to capture 3D chromatin interactions across the genome, allowing chromosome-scale assemblies to be achieved for large eukaryotic genomes^14^.

The karyotype of American mink includes 15 pairs of chromosomes (2n = 30), which is the lowest number of chromosomes among the order *Carnivora*^15^. The first draft of genome assembly for American mink was published in 2017 with a size of 2.4 Gb^16^. However, this draft was highly fragmented and consisted of 7175 scaffolds with the N50 of 6.3 Mb. Despite the advances in genome-wide studies of American mink through the availability of this draft, the lack of chromosomal information as well as existing short-length scaffolds led to some restrictions in genomic studies of mink e.g., linkage disequilibrium^17^, population genomics^3^, genome-wide association studies^18^ and detection of selection signatures^19^. For instance, the analyses of population genomics^3^ and signatures of selection^19^ were restricted to scaffolds >10 Mb due to removing small scaffolds. Consequently, the effective length of genome regions, in which single nucleotide polymorphisms (SNPs) were called, was limited to 802 Mb in the study of homozygous regions across the genome^3^. In addition, it was supposed that existence of small scaffolds prohibited the identification of larger homozygous segments^3^. Furthermore, the annotation of detected genes in these studies were not in the chromosome basis.

The main objective of this study was to design a chromosome-scale genome assembly for American mink using a combination of PacBio long reads, Hi-C technology, and short reads. We compared the quality of newly drafted assembly with the previously available version and explored the gene ontology (GO), gene orthology, demographic history, and synteny of American mink genome in relation to the several close carnivorous species e.g., ferret, otter, dog, and cat.

## Results

### Genome assembly

We used the integration of PacBio long reads, Hi-C sequences and short reads (Table 1) to generate the chromosome-level genome assembly for American mink. Initially, we evaluated the performance of five recommended de novo assemblers for long reads of PacBio. Our results indicated that the Hifiasm draft outperformed the genomes generated by other assemblers in the number of contigs, contiguity, and completeness of genome assembly (Supplementary Table 1). Accordingly, the genome assembly generated by Hifiasm was chosen for further analyses. This initial genome assembly included 291 contigs with the total length of 2.68 Gb. In addition, the average length of contigs was 9.2 Mb with the contig N50 of 39 Mb and the largest contig of 126 Mb. Furthermore, we used the short reads generated at Beijing Genomics Institute (BGI) to polish 80,880 substitution errors in the initial assembly with the final consensus quality of 99.99%.

**Table 1:** The sequencing technologies used for generating the American mink genome assembly.

Sequences	Company	Average length	Number	Coverage rate
High-Fidelity (HiFi) reads	PacBio—Sequel II System	20 Kb	2,884,047	21X
Hi-C reads	PHASE Genomics	150 bp	1,020,932,532	57X
Short reads	BGI Genomics	100 bp	1,021,922,836	38X

The total number of reads generated by each technology was provided along with their average length and coverage rate.

**Fig. 1:**
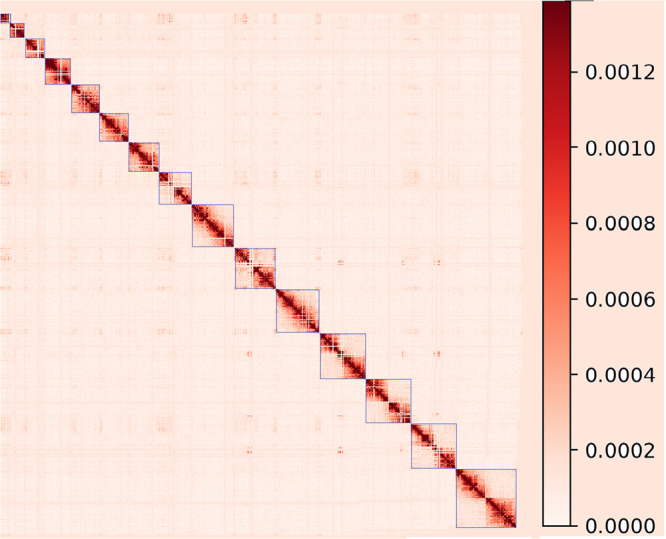
The Hi-C contact map of the assembled American mink genome. The blue squares represent the 15 chromosomes of American mink. The color bar at the right represents the density of Hi-C interactions.

The Hi-C reads were then used to cluster the initial contigs into 15 large scaffolds representing chromosomes of American mink (Fig. 1). Incorporating the Phase Genomics Hi-C reads improved the N50 of the initial draft by more than 5.6 fold. The final genome assembly included 183 scaffolds with the scaffold N50 of 220 Mb. This gap-free genome assembly provided the average scaffold size of 146.5 Mb and the total length of 2.68 Gb. In addition, linkage maps^20,21^ were used to assign the chromosome numbers to corresponding scaffolds (Supplementary Table 2). Out of a total of 157 markers, 136 markers (~87%) were uniquely mapped to autosomal chromosomes. Other markers (21) were mapped to multiple genomic regions and removed from the analysis. In addition, among 32 clone markers, 26 markers (81%) were uniquely assigned to chromosomes. Finally, all sex-linked markers were correctly aligned to chromosome X. Chromosome one was the largest scaffold with the length of 317 Mb whereas chromosome 14 had the lowest length (46.7 Mb). A very high correlation of 0.99 was observed between the physical lengths (bp) and linkage group sizes (cM) of the assigned chromosomes. This genome draft presents a chromosome-level assembly for American mink in which the chromosomes counted for more than 98.6% of the whole genome. The total length of the first six and 12 larger scaffolds occupied 50% and 90% of the whole genome, respectively, indicating high level of continuity.

### Estimation of genome assembly size

The total number of 84,086,112,936 effective 17-mers individuals and 1,378,878,102 effective 17-mers species were counted for short reads with the major coverage rate of 32X (Supplementary Fig. 1). The genome size of 2.63 Gb was estimated for American mink using the 17-mers distribution of short reads, which was comparable to the size of 2.68 Gb obtained through the long-reads assembly.

### Assessment of assembly quality

The final genome assembly was compared to a conserved set of 9226 Benchmarking Universal Single-Copy Orthologs (BUSCOs) from mammalian_odb10 dataset to assess the completeness of the genome (Supplementary Fig. 2). The BUSCO analysis indicated a total of 8899 groups out of 9226 (96.5%) were completely found in our genome draft and only 95 (1%) and 232 (2.5%) BUSCOs were fragmented and missing, respectively.

Table 2 provides the comparison of statistics of two genome assemblies for American mink including the NNQGG.v01 and the ASM_NN_V1. Compared to the first draft, a remarkable reduction was observed in the number of scaffolds for ASM_NN_V1 (183 versus 7175). Furthermore, the total length of genome was increased to 2.68 Gb, which was ~234 Mb longer than the previous version.

**Table 2:** Comparison of genome statistics of two American mink (*Neogale vison*) genome assemblies.

Measure	ASM_NN_V1	NNQGG.v01
Total bases (bp)	2,681,215,271	2,447,189,291
Number of scaffolds	183	7175
Scaffold N50 (bp)	220,349,319	6,814,223
Scaffold L50	6	103
Scaffold N90 (bp)	131,682,864	1,076,351
Scaffold L90	12	430
Longest scaffold (bp)	317,036,279	40,310,764
GC content (%)	42.26	41.60
Repetitive regions proportion (%)	37.42	31
Number of annotated genes	25,377	21,053

The ASM_NN_V1 was generated in the current study and the NNQGG.v01 was the first draft of mink genome published in 2017.

### Read mapping

The alignment rates were estimated for sequencing reads available from the current study along with the reads retrieved from the NCBI database for American mink (NNQGG.v01 assembly version), ferret, otter, and dog (Supplementary Table 3). High mapping percentages of 99.57, 98.46, and 99.96% were observed for reads generated in this study using BGI, Hi-C, and PacBio systems, respectively. In addition, 98.66% of short reads derived from the NNQGG.v01 were mapped to our genome assembly where 94% of paired-end reads were properly paired to this draft. Furthermore, the short reads of ferret (92.57%) and otter (92.63%) were highly mapped to our genome draft whereas the smaller rate of 75.91% was observed for dog genome sequences. The paired-end reads were properly aligned to the ASM_NN_V1 with the percentages of 77.84, 77.53, and 52.54 for otter, ferret, and dog reads, respectively.

### Genome annotation

**Fig. 2:**
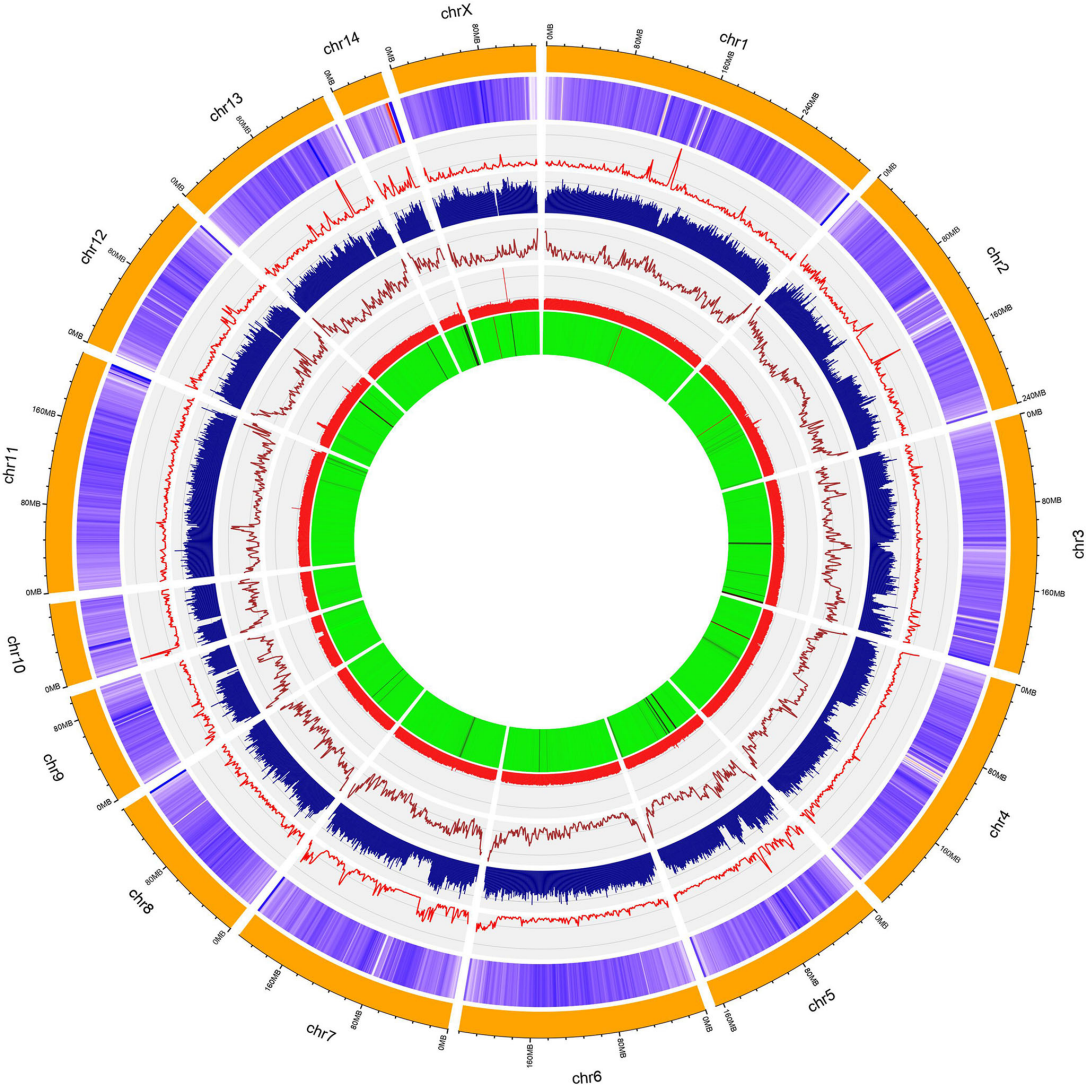
Circos plot representing the 15 chromosomes of American mink genome (ASM_NN_V1). The layers present the heatmap of gene density (purple layer), line plot of SNP density (red layer), histogram of repeat density (blue layer), line plot of GC contents (brown layer), histogram of mapping rate for BGI short reads (pink layer), and heatmap for mapping rate of PacBio reads (green layer). The chromosome lengths were in the Mb unit and the genomic features were plotted for 1 Mb windows.

Table 3 presents the annotation features of American mink genome assembly (ASM_NN_V1) generated through the NCBI Eukaryotic Genome Annotation Pipeline. In total, 25,377 genes were predicted across the American mink genome, which provided an improvement over the number of genes (21,053) reported for the previous mink genome assembly (NNQGG.v01). The mean length of genes was 45.55 Kb with an average of 9.24 exons per gene. In addition, 50,773 RNA transcripts and 44,272 coding DNA sequences (CDSs) were identified across the annotated genome. The BUSCO analysis on the annotated genes indicated that 98.7% of them (96.3% single copy and 2.4% duplicated) could be completely identified in the carnivora_odb10 lineage dataset. In total, 37.42% of the whole genome were identified as repetitive regions and masked by WindowMasker. Figure 2 presents the density of predicted genes, repetitive sequences, number of SNPs, GC content, and alignment rates of 1 Mb windows across the 15 chromosomes of American mink.

**Table 3:** Annotated features of American mink genome assembly (ASM_NN_V1) generated through the NCBI Eukaryotic Genome Annotation Pipeline.

Feature	Number	Mean length (bp)	Min length (bp)	Max length (bp)
Genes	25,377	42,558	55	2,250,073
All transcripts	50,773	3001	55	104,059
mRNA	44,259	3316	105	104,059
misc_RNA	770	2751	100	13,725
tRNA	531	74	62	88
lncRNA	2913	1042	80	24,958
snoRNA	765	107	55	329
snRNA	1234	120	55	199
guide_RNA	25	164	82	411
rRNA	276	790	118	4646
CDSs^a^	44,272	2087	96	103,068
Exons	234,424	274	1	18,102
Introns	211,532	6028	30	1,195,812

^a^Coding DNA sequences

We also used the RepeatModeler to build the de novo repeat library for American mink. The composition of different repetitive elements was retrieved by RepeatMasker based on this de novo repeat library (Supplementary Table 4). The total length of 1,028,367,106 bp was classified as families of repeats covering 38.35% of the whole genome. The LINEs (22.89%) and long terminal repeat elements (5.18%) were the most frequent elements identified in the genome.

### Gene ontology

**Fig. 3:**
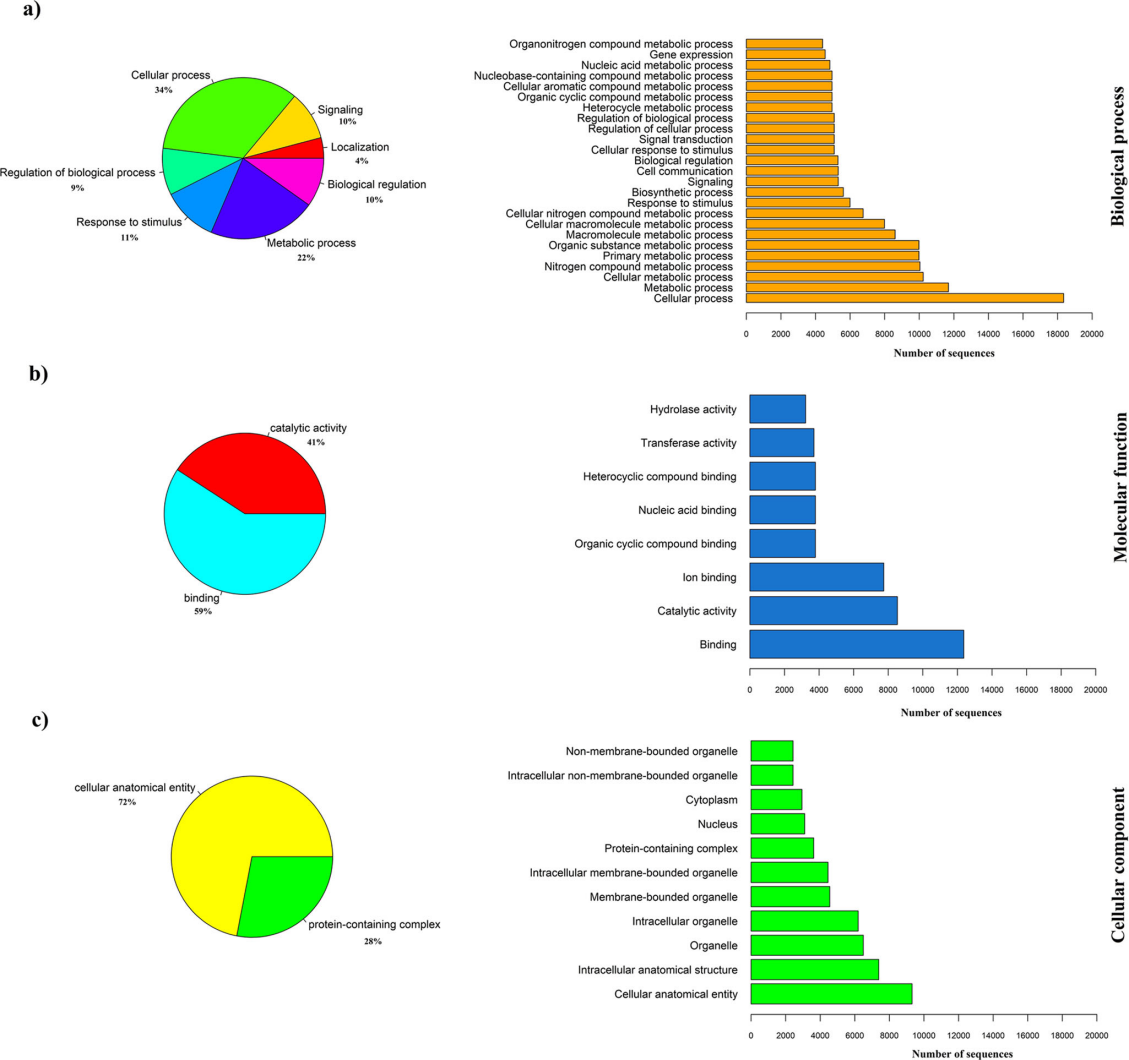
The gene ontology (GO) classification of functionally annotated genes for American mink genome assembly. The distributions of GO terms are presented within the three main GO domains including **a** biological process, **b** molecular function, and **c** cellular component. The bar plots (right side) represent the GO classes across all levels and the pie charts (left side) present level 2 of GO classes.

The proteins predicted by the NCBI eukaryotic gene prediction tool were functionally annotated using the Blast2GO pipeline. The total number of studied protein sequences was 44,272 of which 43,969 sequences were able to blast to the UniprotKB/Swiss-Prot database. Collectively, 34,820 (~79%) out of 44,272 protein sequences were functionally annotated to gene ontology (GO) terms. Among these sequences, 31,211 sequences could map to the gene ontology annotation database (Supplementary Fig. 3). Considering the overlaps among the GO domains, 61% (21,330), 84% (29,260), and 42% (14,635) of the functionally annotated genes belonged to biological process, molecular function, and cellular component, respectively. The distributions of GO terms within three main GO domains including biological process, molecular function, and cellular component are presented in Fig. 3. The cellular process (34%) and metabolic process (22%) were the top two dominant GO terms for level 2 of biological process classes. In addition, the cellular anatomical entity (72%) and protein-containing complex (28%) were the main GO classes found for cellular component domain while binding (59%) and catalytic activity (41%) were the major GO classes involved in the level 2 of molecular function GO terms (Fig. 3 and Supplementary Data 1). These functions might be related to the genome characteristics of mink.

In addition, we used the BLAST analysis to reveal the genomic positions of two major genes involved in the SARS-CoV-2 infection including *ACE2* and *TMPRSS2*. The BLAST analysis indicated that *ACE2* and *TMPRSS2* genes were located on chromosomes X (119,972,697–120,043,088) and 6 (3,983,883–4,017,824), respectively.

### Gene orthology

Comparative genomics was performed on proteomes of American mink along with dog, cat, otter, ferret, and human. Supplementary Table 5 presents the statistics of orthogroups inferred for each species using the OrthoFinder tool. Collectively, 33,561 orthogroups were recognized among the six species, which included 362,242 genes. In addition, 8.6% of genes were included in the species-specific orthogroups (6305). Out of 44,272 genes analyzed from the ASM_NN_V1 draft, 43,293 (97.8%) were presented in the orthogroups. Supplementary Fig. 4 represents the species tree depicted using a concatenated alignment of 2971 single-copy orthogroups. Whereas human was recognized as the outgroup of the tree, the ferret and otter had the closet relationships with American mink. In total, 34,483 orthogroups were inferred at each node in the species tree (hierarchical level). Supplementary Figure 5 shows the Venn diagram of hierarchical orthogroups (HOGs) among the studied species. Whereas the number of American mink-specific HOGs was 171, there were 17,156 HOGs with all species present.

The number of orthologues genes inferred between each pair of studied species are presented in Supplementary Table 6. The American mink had the highest number of orthologues (43,430) with the otter genome whereas the minimum number of orthologues (39,502) were observed with the human genome. Furthermore, Supplementary Table 7 presents the enriched GO terms revealed by the functional annotation of genes clustered into American mink-specific orthogroups. These results indicated that the enrichment of GO terms in the biological processes mainly related to the transport, localization, and biosynthesis activities. The significant GO term in the cellular component domain was related to the nuclear chromosome. In addition, most of the molecular function GO terms were involved in hydrolase and binding activities.

We also made a list of genes previously identified for ecomincally important traits in mink including tolerance to Aleutian disease^19^, body size and pelt length^18^, and fur quality and color^22^. The physical locations of these genes were upgraded based on the new genome assembly (Supplementary Data 2). However, we did not observe any overlapping between these identified genes with mink-specific HOGs.

### Synteny

The dot plots of synteny blocks and rearrangements were depicted between the new genome assembly of mink (ASM_NN_V1) and six other genome assemblies including the first draft of mink genome (NNQGG.v01), ferret, dog, otter, cat, and human (Supplementary Fig. 6). These results revealed the high matches between the first draft of mink genome and the newly introduced genome. Similarly, high identical regions with a few noisy points were observed for alignments of the ferret genome to ASM_NN_V1. Two major rearrangements were observed between chromosomes of the American mink and otter genome. The first one was related to the unique alignments between forward strands inside the chromosome 3 (LR738405.1) of otter genome with chromosome 5 in American mink. The second rearrangements was the reverse alignments found between chromosome 4 of mink and chromosome 4 (LR738406.1) in the otter genome. The synteny pattern of the dog genome included more repetitive and noisy points compared to the other genomes. In addition, more dispersed synteny blocks were observed for the cat and human genomes compared to those found in the ferret and otter. The complete lists of aligned regions between ASM_NN_V1 and other studied genomes are presented in the Supplementary Data 3. The total number of 6, 7, 19, and 62 chromosomal rearrangements (likely fusions/fissions) were observed between the mink genome with otter, cat, human, and dog genomes, respectively. In addition, we identified 15, 19, 41, and 28 inversions between mink with otter, cat, human, and dog genomes, repectively.

Furthermore, large-scale synteny blocks (>10 Kb) were visualized using circos plots (Supplementary Figs. 7–11). A few rearrangements were observed between ASM_NN_V1 and the genomes of ferret and otter. On the other hand, fewer synteny blocks with higher number of rearrangement events were observed for the genome drafts of dog and cat.

### Population-based statistics

We upgraded the population parameters including the average minor allele frequency (MAF), heterozygosity and inbreeding levels based on the excess of homozygosity (F_HOM_) for each color-types sequenced in our previous study^3^ (Supplementary Table 8). The average MAF was 0.198 for all the studied mink with the range of 0.187 (Black-ON i.e., black color type sampled from Millbank Fur Farm) to 0.211 (samples from Canadian Center for Fur Animal Research). The highest observed heterozygosity (30.57%) was observed in Demi whereas the Black-ON had the lowest heterozgosity (27.22%). Furthermore, the highest F_HOM_ was estimated for Black-ON (0.089) with the average of −0.005 among all the studied animals. In addition, we computed the runs of homozygosity (ROH) based on the variants called by ASM_NN_V1 (Supplementary Table 9). The lowest F_ROH_ (with the minimum length of >500 kb) was estimated for Pastel (0.035 ± 0.015) while the highest estimation was observed in Black-ON (0.087 ± 0.044).

### Demographic history

Supplementary Figure 12 presents the demographic history revealed by Pairwise Sequentially Markovian Coalescent (PSMC) analysis for four samples of American mink including the one used to build the genome assembly. Similar demographic trends were observed for all samples validating the output of PSMC. These results indicated a constant decline in effective population size of American mink from approximately two million years ago (MYA) to 100,000 years ago.

## Discussion

The availability of reliable chromosome-level genome assembly is the foundational step to develop genomic studies for different species. In this study, we took advantage of PacBio long reads along with Hi-C technology to achieve a high-quality chromosome-level genome assembly for American mink. The newly assembled genome of mink provided remarkable improvements over the first draft (Table 2). The length of our genome assembly (2.68 Gb) was closer to the estimated genome size of 2.7 Gb suggested for American mink whereas the first draft of genome assembly (NNQGG.v01) provided the length of 2.44 Gb^16,23^. Overall, 234 Mb were added to the length of previously assembled genome, of which 166.35 Mb (71.09%) were in repetitive regions. In addition, 1254 genes were added to the genome annotation of American mink through including the sequences missed from the previous genome assembly.

We also provided more contiguous genome with a smaller number of scaffolds. In addition, the contiguity (220,35 Mb) compares well to other outstanding assembly projects e.g., goat^24^ (ARS1, N50 = 87.27 Mb), human^25^ (GRCh38.p13, N50 = 67.79 Mb), and buffalo^26^ (UOA_WB_1, N50 = 117.21 Mb). We achieved a gap-free assembly in which more than 98.6% of the whole genome was assigned to the chromosomes. Overall, to our knowledge, this is the most continuous de novo genome assembly to date, with chromosome-scale scaffolds and no gaps. Although the chromosome assignments were validated using linkage groups, there are still 168 scaffolds unassigned to specific genomic regions which require further investigation. The chromosome coverage achieved (98.6%) was comparable with those reported for goat^24^ American pika^27^ and buffalo^26^ (88.32%, 97%, and 99%, respectively), which were regarded as some of the superior new reported assemblies.

The sequence libraries of NNQGG.v01 were aligned to ASM_NN_V1 with the mapping rate of 98.66%, which was slightly higher than the realignment rate of 98% reported for the NNQGG.v01^16^. Furthermore, the alignment rate of short reads from ferret (92.57%) to ASM_NN_V1 was comparable to those observed (86.42–95.90%) for NNQGG.v01^16^. The lower rate of 75.91% for alignment of genome sequences of dog to mink genome might be due to higher evolutionary distances between these species. In addition, The BUSCO completeness of 96.5% for ASM_NN_V1 was slightly higher than the rate of 95.8% observed for NNQGG.v01^16^. Consistent with these results, slight improvements were also reported for the BUSCO completeness of PacBio-based assemblies in buffalo (0.6%)^26^, zebra finch, and hummingbird (1–3%)^28^ in comparison with the short-read-based assemblies.

The GC content of ASM_NN_V1 (42.26%) was comparable to that observed for NNQGG.v01 (41.60%). However, higher proportion (38.35%) of de novo repetitive regions was estimated for ASM_NN_V1 compared to the rate of 31% reported for NNQGG.v01. Cai et al. (2017)^16^ pointed out that the real repeats content of NNQGG.v01 was likely larger since this draft had 10% fewer sequences compared to the estimated genome size. The higher rates of 42.96% and 43% were reported for repetitive genome regions in dog (NCBI Canis lupus familiaris Annotation Release 105) and cat^29^ genomes, respectively. Similarly, less repeat content was estimated for NNQGG.v01 compared to dog and cat genomes suggesting that dog and cat likely have more repeated regions compared to American mink^16^. Although the LINEs constituted the highest proportion of repeated regions in both the ASM_NN_V1 (22.89%) and NNQGG.v01 (14.76%), the new estimation is in accordance with the rates reported in the four *Caniformia* genomes including panda (20.90%), polar bear (21.77%), dog (20.84%), and ferret (19.42%)^30^. Furthermore, the proportions estimated for the long terminal repeat elements in panda (5.40%), polar bear (5.51%), dog (4.95%), and ferret (4.73%) were consistent with the percentage of 5.18 observed for the ASM_NN_V1. However, the SINEs represented a smaller proportion of repeat content (1.1%) in comparison with 7.05% reported for NNQGG.v01. This disagreement might be due to the differences in the length of the genomes and resources included in the repeat libraries. Similar large differences were observed between the percentages of LINEs and SINEs estimated for genome assemblies of Tarim red deer^31^ (37.76% versus 4.22%), Takifugu bimaculatus (12.31% versus 0.29%)^32^, and Tibetan black bear^33^ (25.02% versus 6.25%).

In total, 4324 more genes were predicted through the NCBI Eukaryotic Genome Annotation Pipeline for ASM_NN_V1 compared with the NNQGG.v01. In addition to the predicted genes, the genome annotation provided the complementary information on all transcripts, CDSs, exons, and introns (Table 3). This update of genome annotation provides a more comprehensive picture of genomic characteristics in American mink.

Moreover, 22,536 orthologous gene families were identified for American mink that included 67.1% of all identified orthogroups among the studied species. The rooted gene trees were used to infer the orthogroups at each hierarchical level because it was suggested that the inference approach was more accurate than the gene similarity-based methods^34^. American mink shared the greatest number of orthologues with ferret and otter, indicating their evolutionary relationship. The GO annotation of genes in the American mink-specific HOGs revealed their functional roles in biosynthesis, localization, and transportation of protein and nitrogen-containing substances, implying that they might cause unique characteristics to the American mink genome. Compared to ASM_NN_V1, 14,066 orthologous gene families containing 17,052 genes were reported for the NNQGG.v01, implying an outstanding improvement in the gene orthology of the newly drafted genome assembly. No overleaping genes were identified between the previous publications and the genes clustered into American mink-specific orthogroups implying that more genome association analyses would be required to enrich the gene ontology for this species.

The susceptibility of American mink to SARS-CoV-2 has been demonstrated in several studies indicating widespread economic loss across the mink industry^10,35,36^. It seems that the receptor for SARS-CoV-2 in the mink is comparable to that in humans enabling the virus to transmit from mink to human^37^. Angiotensin-converting enzyme 2 (*ACE2*) and type 2 transmembrane serine proteases (*TMPRSS2*) are recognized as two main genes involving in molecular mechanisms of virus entrance to human cells^38^. SARS-CoV-2 uses *ACE2* to attach to the human cells through its receptor-binding domain. In addition, the *TMPRSS2* plays critical role in cleaving the spike (S) protein, and facilitating viral activation and cell entry^39,40^. Our genome annotation and BLAST analysis revealed that *ACE2* gene was located on chromosome X (119,972,697–120,043,088) in American mink genome, which is comparable to its chromosomal location in human genome (chr X: 15,518,197–15,607,211) with a percentage identity of 88.41. The *TMPRSS2* was annotated on chromosome 6 (3,983,883–4,017,824) and chromosome 21 (41,464,305–41,508,158) in American mink and human genomes, respectively, which was in accordance with the synteny and alignment analyses of these genomes (Supplementary Fig. 6 and Supplementary Data 3). Accordingly, American mink could be a suitable animal model to investigate the viral fitness, vaccine efficacy, reinfection, and contagiousness of virus mutants^10,41^. The availability of high-quality reference as well as genome annotation could facilitate the efforts to understand the SARS-CoV-2 mechanisms in the American mink and potentially humans. However, more experimental studies on the virology aspect of SARS-CoV-2 would be required.

A few rearrangements were found between ASM_NN_V1 and the genomes of ferret and otter (Supplementary Fig. 6 and Supplementary Data 3). The highly contiguous structure of ASM_NN_V1 facilitated the detection of rearrangements between species whereas the fragmented sequences of NNQGG.v01 led to the restrictions in the genome alignment and synteny analyses performed on dog and ferret genomes^16^. However, more investigations are suggested in order to find out the structure of chromosomes e.g., the centromeric regions and segmental duplications.

The demographic analysis revealed an initial decline in the effective population sizes beginning approximately two MYA, which might be related to the emergence of New World American mink and is supported by the first fossil records of the occurrence of New World American mink dating back to early Pleistocene (~1.9 MYA)^42^. It seems that the Pliocene epoch (5.3–1.8 MYA) was the span time in which the widespread differentiation occurred between *Mustela* species including all the American species. This interval gave rise to the divergence of New World American mink (*Mustela vison*, the former name for *Neogale vison*) and long-tailed weasel (*Mustela frenata*) from the other *Mustela* species^43^. Furthermore, the fossil evidence dating to North American Pleistocene indicated the split between *Mustela vison* and *Mustela frenata* at this epoch^42,44,45^. However, Harding and Smith (2009)^44^ estimated an older time (5–6.1 MYA) for the divergence of American mink and its smaller weasel relatives. These differences were attributed to the taxonomic sampling, genetic samples, inference methodologies, and fossil calibrations applied in the studies.

However, the interpretation of historical population sizes obtained by PSMC method should be conservative owing to the possible distortion of coalescent patterns. It was shown that there was variation in the results obtained for individuals from the same species by PSMC method e.g., those were reported for lion and black-and-white Ficedula flycatchers^46,47^. These variations can be due to the weakness of the software’s algorithm causing bias in identifying the heterozygous sites and make it difficult to infer the historical *Ne* using this approach^46^.

Our study confirmed the importance of high-quality genome from the same species to infer the evolutionary events and population biology. This continuous draft allowed us to explore the recent inbreeding and longer homozygous segments across the genome, which was not possible previously^3,17^. The averages of MAF (0.198 versus 0.216) and heterozygosity (30% versus 30.45%) were decreased whereas higher F_HOM_ (−0.006 versus −0.166) was observed in comparison with the values obtained using NNQGG.v01. Although the number of detected homozygous segments was increased using the ASM_NN_V1 in comparison with the previous study^3^ (on average 103 versus 82 per individual for ROH > 500 kb), the averages of F_ROH_ were decreased among the studied populations (on average 0.043 versus 0.097 for ROH > 500 kb). In addition, we could identify longer ROH (>4 Mb) using the data from the new genome assembly whereas only ROH > 2 Mb were detected in our previous study^3^. These results provided more accurate estimations due to including the whole length of the genome in the computations. Furthermore, longer ROH (>4 Mb) can reflect a better picture of recent inbreeding in the populations. These analyses could be critical for the conservation biology of American mink, particularly in European countries where this animal has been recognized as an invasive species^48^.

The availability of a reliable chromosome-level genome assembly would support efforts to develop genome-enabled selective breeding programs and contribute to the basic understanding of the American mink genome. The contiguous chromosome-level genome assembly supports SNP discovery and design of high-throughput SNP panels, which can facilitate genome-wide association studies and genomic selection in American mink. Moreover, a reliable genome assembly is a critical part of gene expression, epigenetics, and whole-genome genotyping analyses^49^.

## Methods

### Ethics declarations

All protocols were approved by the Dalhousie University Animal Care and Use Committee (certification numbers: 2018-009 and 2019-012), and mink used in this study were cared for according to the Code of Practice for the Care and Handling of Farmed Mink guidelines (https://www.nfacc.ca/pdfs/codes/mink_code_of_practice.pdf).

### Sample, DNA extraction, and sequencing

The target sample for this study was selected based on genetic structure analyses of whole-genome sequences (with the average coverage rate of ~36×) of 100 American mink from two Canadian farms^3^. A non-admixed black mink with the highest level of homozygosity was chosen for genome assembly. Accordingly, the tongue tissue of an adult female mink from the commercial mink farm of Millbank Fur Farm (Rockwood, ON, Canada) was used for genome assembly.

DNA isolation was conducted using MagAttract HMW DNA Kit (QIAgen, Hiden, Germany) by Bio S&T Inc (Québec, Canada). After passing the quality controls, the DNA was used to construct the PacBio libraries following the manufacturer’s instructions. Three libraries were prepared and sequenced using the PacBio Sequel II SMRT platform at the Génome Québec (Québec, Canada). Collectively, 2,884,047 reads were generated with the average size of ~20 Kb and the genome coverage rate of ~21X.

In addition, cells were extracted following the Phase Genomics protocols and were then sent to Phase Genomics (Seattle, WA, USA) to prepare the Hi-C library. The library was constructed using the fragments produced by the restriction enzyme *DPNII*. After that, the library was sequenced on the Illumina HiSeq 4000 generating 1,020,932,532 reads with the size of 150 bp, which included 760,556,698 paired-end reads.

Genomic DNA for short-read sequencing was extracted using DNeasy Blood and Tissue Kit (Qiagen, Hilden, Germany) according to the manufacture protocol. The DNA was then sequenced to produce 100 bp paired-end reads using the BGISEQ-500 platform (BGI, Guangdong, China). The adaptors and low-quality sequences were filtered using SOAPnuke software^50^. In total, 1,021,922,836 read pairs with the size of 100 bp were generated, which provided physical coverage of ~38X.

### Estimation of the genome size

Genome size was estimated based on the k-mer approach as implemented in gce-1.0.2^51^. In total, 1,021,922,836 paired-end reads with the size of 100 bp were used to estimate k-mer distribution (k = 17). A major peak was observed at 32X for the 17-mers distribution (Supplementary Fig. 1). The genome size was estimated as the number of k-mers divided by the peak depth of the reads.

### Genome assembly

The initial genome assembly was built using the HiFi long reads from the PacBio system. We compared the performance of five de novo assemblers including WTDBG2^52^, Flye^53^, Hifiasm^54^, Hicanu^55^, and IPA (https://github.com/PacificBiosciences/pbbioconda/wiki/Improved-Phased-Assembler) to generate the continuous and complete assembly. Assessment of contiguity (N50), overall size and Benchmarking Universal Single-Copy Orthologs (BUSCO) completeness of genome drafts revealed that the Hifiasm outperformed the other assemblers (Supplementary Table 1). At the next step, the genome assembly was corrected using the BGI short reads as implemented in the Polca software^56^. Subsequently, the Hi-C reads were mapped to the initial draft derived from the previous steps to create the 3D-genome structure. The Hi-C reads were aligned to the genome assembly using the minimap2^57^ to generate a contact map as implemented in the hicstuff library^58^. After that, the contigs were scaffolded and polished using the instaGRAAL^59^ program to obtain a chromosome-level assembly. The level (resolution) was set to be five and default settings were used to generate a contact map. The final contact map was visualized using the hicstuff library.

We then used the linkage maps^20,21^ previously published for American mink to validate the chromosome assignments achieved by the Hi-C contact map. Collectively, the fasta files of 157 microsatellite markers as well as 32 clones containing microsatellite markers by in situ hybridization^21^ (of which 18 markers were shared with the initial 157 markers) were downloaded from the NCBI site and aligned to the genome assembly using GraphAligner^60^. Furthermore, six sex-linked markers were used to validate the X chromosome^20^. The minimum identity percentage of 90% was considered to assign markers to relevant genomic regions. The markers with multiple alignments were discarded from the dataset.

### Assessment of assembly quality

The assembly-stats v1.0.1^61^ was used to compute the number of scaffolds, average of scaffold lengths, number of gaps, L50, N50, and total bases assembled by different de novo assemblers. Furthermore, the completeness of genome assemblies was assessed using BUSCO v5.2.2^62^ with mammalia_odb10 lineage dataset including 9226 Benchmarking Universal Single-Copy Orthologs. The BUSCO was run under the genome mode to compute the proportion of complete, fragmented, and missing genes across the dataset.

### Read mapping

To evaluate the mapping rate of reads, the BGI short reads were realigned to genome assembly using BWA-MEM^63^. Subsequently, SAMtools^64^ was used to compute the mapping percentage and the depth of aligned reads. Likewise, Hi-C short reads were aligned to the genome assembly. In addition, we realigned the PacBio HiFi reads to the assembly using Minimap2^57^ to assess the mapping rates. Moreover, the short reads available from the first draft of the mink genome (ERR1676595 to ERR1676601) were downloaded from the NCBI and aligned to the new genome draft (ASM_NN_V1) to validate the correctness of genome assembly. Finally, we used the short reads available through the NCBI for other carnivores including ferret (SRR085064, SRR085066, SRR085080, and SRR085081), otter (ERR3316145, ERR3316146, and ERR3316147), and dog (SRR12588476) to assess the genome alignment rates.

### Genome annotation

The assembled genome of American mink was annotated through the NCBI Eukaryotic Genome Annotation Pipeline^65,66^. This pipeline automates all steps of genome annotation including detection and masking repetitive regions using WindowMasker^67^, alignment of experimental evidence (transcripts, RNA-Seq, and proteins), prediction of genes via model-based and ab initio procedures, and mapping of curated genes. BUSCO was run in “protein” mode on annotated genes using the carnivora_odb10 lineage dataset to assess the completeness of final annotation.

### Gene ontology

Blast2GO (B2G) v6.0.3^68^ was used to functionally annotate the predicted genes by the NCBI Eukaryotic Genome Annotation Pipeline. The functional annotation of protein-coding sequences from the American mink was queried using BLASTP v2.2.26^69^ against UniprotKB/Swiss-Prot database 2021_03 release with an E-value cutoff of 10^−5^. The associated GO terms were then mapped to BLAST hits using Blast2GO. Furthermore, the InterProScan^70^ was used to map GO terms to protein sites, families, repeats, and domains available through the InterPro member databases using the default settings in Blast2GO. Finally, the GO terms from InterProScan were merged to those queried by BLAST in the previous step.

### Gene orthology

We also used the protein-coding sequences from the American mink, otter, ferret, dog, cat, and human to construct gene families (orthogroups) using OrthoFinder v2.5.4^34^. DIAMOND^71^ was used as the sequence search program to infer orthogroups. The sequence similarity and clustering steps were performed using the Markov cluster (MCL) algorithm^72^. The MAFFT^73^ was then implemented as the multiple protein sequence aligner and the FastTree2 was used for maximum likelihood gene trees inference^74^. A concatenated alignment of single-copy orthogroups was then used to construct the species tree with FastTree2. The human species was chosen as the outgroup of the phylogenetic tree according to the OrthoFinder inferences and the Specie Tree Root Inference from Duplication Events (STRIDE) was used for rooting the trees. The orthogroups were then inferred at each node in the species tree by analyzing the rooted gene trees.

The genes clustered into American mink-specific orthogroups (those that were not found in other species) were used to perform functional enrichment analysis. Fisher’s exact test was used in combination with False Discovery Rate (FDR) correction for multiple testing with the significant threshold of 0.05 as implemented in the Blast2GO tool. The entire GO annotation of American mink was used as the reference set.

Furthermore, we mapped the known genes related to fur quality and fur color^22^, Aleutian disease^19^ and body size and pelt length^18^ to newly assembled genome. The physical location of these genes was upgraded based on chromosomes information available from ASM_NN_V1.

### Synteny

The new genome assembly of American mink was aligned to the whole-genome sequences of the first mink assembly (NNQGG.v01), ferret, dog, otter, cat, and human using the nucmer program within MUMmer^75^ (v.4.0.0beta2) to reveal syntenic blocks and rearrangements. The nucmer program was run with the default parameters and the summary information was presented and filtered using show-coords with the parameters “-o -l -r -I 98 -L 1000”. Alignments of two whole genomes were displayed using the Dot program (Dot: Interactive dot plot for genome-genome alignments (sandbox.bio). Furthermore, large-scale (>10 Kb) synteny blocks between whole genomes were visualized using circos assembly consistency (Jupiter) plot pipeline (JustinChu/JupiterPlot: A Circos-based tool to visualize genome assembly consistency (github.com). The ASM_NN_V1 was used as the reference genome and the pipeline was run using default settings.

### Population-based statistics

The whole-genome sequences of 100 mink were used to provide the population-related parameters including MAF, heterozygosity, and genomic inbreeding rates (F_HOM_ and F_ROH_). The samples were collected from two farms including the Canadian Center for Fur Animal Research (CCFAR) at Dalhousie Faculty of Agriculture (Truro, NS, Canada) and Millbank Fur Farm (Rockwood, ON, Canada) and were previously studied for aforementioned parameters based on the analyses performed on NNQGG.v01^3^. We used the newly genome assembly to update theses population-based parameters. The ASM_NN_V1 was used as the reference for alignment and variant calling steps^3^. The F_ROH_ were then computed using the following parameters: minimum window size of 20 SNPs, genotyping error rate of 0.01 and sliding window step size of one SNP^3^. The total length of genomic regions in which SNPs could be called, was upgraded to 2.68 Gb. The minimum length of ROH was set to be 500 kb, 1 Mb, 2 Mb, and 4 Mb at different runs.

### Demographic history

We used the PSMC model^76^ to explore the historical variations in effective population sizes of American mink. The short reads were mapped to the ASM_NN_V1 using BWA-MEM^63^ and consensus sequences were called using SAMtools mpileup^64^ and BCFtools call. The VCF file was converted to a fastq-like format using vcfutils “vcf2fq”, excluding the sites with a minimum read depth <10 and maximum depth >100. Subsequently, consensus sequences in fastq-like format were converted into PSMC fasta using “fq2psmcfa” removing blocks with more than 20% missing data (https://github.com/lh3/psmc). The PSMC program was tested with several values for -t and -p parameters as those were applied for the *Felidae*^77^, bat-eared fox and aardwolf^78^, great ape^79^, pied flycatcher, and collared flycatcher^80^. Finally, the parameters -t15 -r4 -p “4 + 10*3 + 4” were chosen for this study, and the computations were bootstrapped 100 times to estimate the variance in ancestral population sizes. The mutation rate of 1.0e − 08 (mutation/site/generation)^78,81,82^ and the generation time of four years^83,84^ were applied to visualize the PSMC graph using “psmc_plot.pl” script. To validate the results, the same analysis was performed on three additional samples including a black mink from the same farm and two pastel and black mink from the Canadian Centre for Fur Animal Research (CCFAR) at the Dalhousie Faculty of Agriculture (Truro, NS, Canada).

### Statistics and reproducibility

The functional enrichment analysis was performed using the Blast2GO tool^68^. The statistical significance of GO terms was evaluated using Fisher’s exact test in combination with FDR correction for multiple testing (*P* < 0.05). A black mink with the highest level of homozygosity was selected to generate the genome assembly. This sample was chosen based on the analyses of whole-genome sequences of 100 American mink from two Canadian farms^3^.

### Reporting summary

Further information on research design is available in the Nature Research Reporting Summary linked to this article.

## Supplementary information

Supplementary information

Description of Additional Supplementary Data

Supplementary Data 1

Supplementary Data 2

Supplementary Data 3

Reporting Summary-New

## Data Availability

Source data underlying Fig. 3 are presented in Supplementary Data 1. Raw reads for genome sequencing have been deposited at NCBI Short Read Archive (SRA) under the accession number SRX11368813 to SRX11368817. This Whole Genome Shotgun project has been deposited at DDBJ/ENA/GenBank under the accession JAIAWX000000000. The version described in this paper is version JAIAWX010000000. The genome assembly has been deposited at the NCBI under BioProject number PRJNA741394. The accession numbers of SRA files used in the analyses of other species were mentioned in the Methods section.
