# Supplementary material for: A chromosome-level genome assembly reveals genomic characteristics of the American mink (Neogale vison)
Source: Commun Biol. 2022 Dec 16;5:1381. doi: 10.1038/s42003-022-04341-5 (PMC9757699; doi:10.1038/s42003-022-04341-5)
Supplement: Supplementary file 1 — Supplementary information [file 42003_2022_4341_MOESM1_ESM.pdf]

# A chromosome-level genome assembly reveals genomic characteristics of the American mink (*Neogale vison*)

**Karim Karimi [1], Duy Ngoc Do [1], Jingy Wang [1], John Easley [2, 3], Shima Borzouie [1], Mehdi Sargolzaei [4, 5], Graham Plastow [6], Zhiquan Wang [6] & Younes Miar [1, *]**

[1] Department of Animal Science and Aquaculture, Dalhousie University, Truro, Nova Scotia, Canada

[2] Joint Mink Research Committee, Fur Commission USA, Preston, Idaho, USA

[3] Mink Veterinary Consulting and Research Service, Plymouth, Wisconsin, USA

[4] Department of Pathobiology, University of Guelph, Guelph, Ontario, Canada

[5] Select Sires Inc., Plain City, Ohio, USA

[6] Livestock Gentec, Department of Agricultural, Food and Nutritional Science, University of Alberta, Edmonton, Alberta, Canada

* Corresponding author

Email: miar@dal.ca

**Supplementary Table 1 Total length, number of contigs, N50, and completeness of genome drafts built using five *de novo* assemblers recommended for PacBio long-reads.**

| De novo Assembler | Total length (Gb) | Number of contigs | N50 (Mb) | BUSCO completeness | | | |
|---|---|---|---|---|---|---|---|
| | | | | Complete | | Fragmented | Missing |
| | | | | Single copy | Duplicated | | |
| Hifiasm | 2.68 | 291 | 39 | 94.7% | 1.5% | 1.1% | 2.7% |
| WTDBG2 | 2.43 | 2,731 | 19.33 | 91.7% | 1.3% | 2.1% | 4.9% |
| Flye | 2.66 | 3,529 | 18.28 | 95% | 1.4% | 1% | 2.6% |
| IPA | 2.96 | 1,496 | 9.71 | 88% | 8.5% | 1% | 2.5% |
| Hicanu | 4.83 [1] | 15,541 | 1.55 | 69.1% | 27.8% | 0.9% | 2.2% |

[1] Hicanu generated a diploid genome assembly.

**Supplementary Table 2 The chromosome IDs along with the number and percentage of uniquely assigned markers to corresponding scaffold IDs.**

| Scaffold ID | Chromosome ID | Length (bp) | Number of uniquely assigned markers | Percentage of uniquely assigned markers |
|---|---|---|---|---|
| 110 | 1 | 317,036,279 | 24 | 96 |
| 109 | 2 | 240,416,976 | 14 | 100 |
| 108 | 3 | 235,645,773 | 16 | 100 |
| 107 | 4 | 231,359,643 | 14 | 100 |
| 103 | 5 | 167,246,402 | 7 | 100 |
| 106 | 6 | 224,559,537 | 15 | 100 |
| 105 | 7 | 207,076,058 | 9 | 64 |
| 100 | 8 | 144,012,018 | 5 | 100 |
| 98 | 9 | 101,698,841 | 3 | 100 |
| 97 | 10 | 75,573,270 | 8 | 89 |
| 104 | 11 | 220,349,319 | 4 | 67 |
| 101 | 12 | 148,690,698 | 7 | 88 |
| 102 | 13 | 152,771,447 | 8 | 100 |
| 96 | 14 | 46,742,321 | 2 | 100 |
| 99 | X | 131,682,864 | 6 | 100 |
| Total | - | 2,644,861,446 | 142 | 93.5 |

**Supplementary Table 3 The realignment rate of sequence reads to ASM_NN_V1.** The reads generated for constructing two American mink genome assemblies (ASM_NN_V1 and NNQGG.v01) were used along with the reads derived from the ferret, otter, and dog genomes.

| **Resource** [1] | **Sequencing platform** | **Length** | **Total number** | **Mapping percentage** | **Properly paired (%)** | **Physical coverage (x)** |
|---|---|---|---|---|---|---|
| ASM_NN_V1 | BGI short reads | 100 bp | 1,021,922,836 | 99.57 | 97.5 | 38 |
| | Hi-C short reads | 150 bp | 1,020,932,532 | 98.46 | 38.6 | 57 |
| | PacBio long reads | 20 Kb | 2,884,047 | 99.96 | - | 21 |
| NNQGG.v01 | Illumina short reads | 100 bp | 3,409,536,700 | 98.66 | 94 | 127 |
| Ferret | Illumina short reads | 100 bp | 753,661,630 | 92.57 | 77.53 | 28 |
| Otter | Illumina short reads | 150 bp | 634,627,003 | 92.63 | 79.84 | 35 |
| Dog | Illumina short reads | 150 bp | 1,956,571,742 | 75.91 | 52.54 | 109 |

[1] Sequencing reads of ferret (SRR085064, SRR085066, SRR085080, and SRR085081), otter (ERR3316145 to ERR3316147) and dog (SRR12588476) were derived from MusPutFur1.0, mLutLut1.2 and ROS_Cfam_1.0 assembly projects, respectively. The reads of first mink genome assembly (NNQGG.v01 assembly version) were downloaded through the NCBI database using the SRA numbers from ERR1676595 to ERR1676601.

**Supplementary Table 4 The composition of repetitive elements identified across the American mink genome assembly based on the *de novo* repeat library built in RepeatModeler.**

| Family | | | Number of elements | Length (bp) | Percentage of genome |
|---|---|---|---|---|---|
| Retroelements | | | 2,774,661 | 782,100,171 | 29.17 |
| | SINEs | | 259,731 | 29,509,563 | 1.10 |
| | Penelope | | 0 | 0 | 0 |
| | LINEs | | 1,834,956 | 613,740,802 | 22.89 |
| | | CRE/SLACS | 0 | 0 | 0 |
| | | L2/CR1/Rex | 111,162 | 18,346,831 | 0.68 |
| | | R1/LOA/Jockey | 0 | 0 | 0 |
| | | R2/R4/NeSL | 0 | 0 | 0 |
| | | RTE/Bov-B | 0 | 0 | 0 |
| | | L1/CIN4 | 1,723,794 | 595,393,971 | 22.21 |
| | LTR elements | | 679,974 | 138,849,806 | 5.18 |
| | | BEL/Pao | 0 | 0 | 0 |
| | | Ty1/Copia | 173 | 165,853 | 0.01 |
| | | Gypsy/DIRS1 | 8,447 | 1,041,636 | 0.04 |
| | | Retroviral | 629,787 | 130,603,629 | 4.87 |
| DNA transposons | | | 213,761 | 38,682,420 | 1.44 |
| | hobo-Activator | | 166,977 | 26,483,851 | 0.99 |
| | Tc1-IS630-Pogo | | 45,986 | 11,804,368 | 0.44 |
| | En-Spm | | 0 | 0 | 0 |
| | MuDR-IS905 | | 0 | 0 | 0 |
| | PiggyBac | | 276 | 135,644 | 0.01 |
| | Tourist/Harbinger | | 0 | 0 | 0 |
| | Other | | 0 | 0 | 0 |
| Rolling-circles | | | 4,895 | 638,797 | 0.02 |
| Unclassified | | | 269,957 | 144,309,438 | 5.38 |
| Total interspersed repeats | | | | 965,092,029 | 35.99 |
| Small RNA | | | 143,639 | 12,452,380 | 0.46 |
| Satellites | | | 63,908 | 5,674,408 | 0.21 |
| Simple repeats | | | 689,160 | 38,915,909 | 1.45 |
| Low complexity | | | 108,118 | 5,593,583 | 0.21 |
| Total | | | | 1,028,367,106 | 38.35 |

**Supplementary Table 5 The statistics of the revealed orthogroups derived from the proteomes of American mink, dog, cat, otter, ferret, and human.**

| Species | Otter | Cat | Dog | Ferret | Human | Mink |
|---|---|---|---|---|---|---|
| Number of genes | 48,337 | 54,726 | 66,768 | 48,107 | 116,263 | 44,272 |
| Number of genes in orthogroups | 47,052 | 52,797 | 63,625 | 46,761 | 108,714 | 43,293 |
| Number of unassigned genes | 1,285 | 1,929 | 3,143 | 1,346 | 7,549 | 979 |
| Percentage of genes in orthogroups | 97.3 | 96.5 | 95.3 | 97.2 | 93.5 | 97.8 |
| Percentage of unassigned genes | 2.7 | 3.5 | 4.7 | 2.8 | 6.5 | 2.2 |
| Number of orthogroups containing species | 23,774 | 23,197 | 23,958 | 22,634 | 26,965 | 22,536 |
| Percentage of orthogroups containing species | 70.8 | 69.1 | 71.4 | 67.4 | 80.3 | 67.1 |
| Number of species-specific orthogroups | 210 | 695 | 1,028 | 379 | 3,826 | 167 |
| Number of genes in species-specific orthogroups | 578 | 2,636 | 4,528 | 1,288 | 22,988 | 646 |
| Percentage of genes in species-specific orthogroups | 1.2 | 4.8 | 6.8 | 2.7 | 19.8 | 1.5 |

**Supplementary Table 6 The number of orthologues (orthologues genes) shared between each pair of the studied species.** Each paired comparison presented the total number of genes in the first species that have orthologues in the second species.

| **Species** | **Otter** | **Cat** | **Dog** | **Ferret** | **Human** | **Mink** |
|---|---|---|---|---|---|---|
| Otter | 0 | 43,050 | 44,103 | 45,007 | 42,465 | 45,361 |
| Cat | 48,043 | 0 | 48,811 | 46,338 | 46,912 | 46,922 |
| Dog | 56,415 | 56,043 | 0 | 54,427 | 54,867 | 55,159 |
| Ferret | 46,301 | 42,357 | 43,339 | 0 | 41,184 | 45,263 |
| Human | 78,702 | 78,703 | 80,522 | 75,865 | 0 | 77,120 |
| Mink | 43,430 | 40,045 | 41,033 | 42,558 | 39,502 | 0 |

**Supplementary Table 7 The enriched genome annotation (GO) terms related to genes presented in the American mink-specific orthogroups.** The significant threshold of 0.05 was used for FDR test. The entire GO annotation of American mink was used as the reference.

| GO ID | GO Name | GO Category | FDR [1] | P-Value |
|---|---|---|---|---|
| GO:0008104 | Protein localization | Biological process | 0.003 | 0.000 |
| GO:0071705 | Nitrogen compound transport | Biological process | 0.003 | 0.000 |
| GO:0071702 | Organic substance transport | Biological process | 0.003 | 0.000 |
| GO:0045184 | Establishment of protein localization | Biological process | 0.003 | 0.000 |
| GO:0015031 | Protein transport | Biological process | 0.003 | 0.000 |
| GO:0006790 | Sulfur compound metabolic process | Biological process | 0.003 | 0.000 |
| GO:0033036 | Macromolecule localization | Biological process | 0.003 | 0.000 |
| GO:0030705 | Cytoskeleton-dependent intracellular transport | Biological process | 0.021 | 0.000 |
| GO:0042254 | Ribosome biogenesis | Biological process | 0.042 | 0.001 |
| GO:0006518 | Peptide metabolic process | Biological process | 0.042 | 0.003 |
| GO:0006412 | Translation | Biological process | 0.042 | 0.003 |
| GO:1901566 | Organonitrogen compound biosynthetic process | Biological process | 0.042 | 0.003 |
| GO:0043043 | Peptide biosynthetic process | Biological process | 0.042 | 0.003 |
| GO:0043604 | Amide biosynthetic process | Biological process | 0.042 | 0.003 |
| GO:0043603 | Cellular amide metabolic process | Biological process | 0.042 | 0.003 |
| GO:0007267 | Cell-cell signaling | Biological process | 0.045 | 0.004 |
| GO:0000228 | Nuclear chromosome | Cellular component | 0.020 | 0.000 |
| GO:0008289 | Lipid binding | Molecular function | 0.003 | 0.000 |
| GO:0019843 | rRNA binding | Molecular function | 0.012 | 0.000 |
| GO:0003924 | GTPase activity | Molecular function | 0.042 | 0.003 |
| GO:0016787 | Hydrolase activity | Molecular function | 0.042 | 0.003 |
| GO:0016462 | Pyrophosphatase activity | Molecular function | 0.042 | 0.003 |
| GO:0016817 | Hydrolase activity, acting on acid anhydrides | Molecular function | 0.042 | 0.003 |
| GO:0016818 | Hydrolase activity, acting on acid anhydrides, in phosphorus-containing anhydrides | Molecular function | 0.042 | 0.003 |
| GO:0017111 | Nucleoside-triphosphatase activity | Molecular function | 0.042 | 0.003 |

[1] False Discovery Rate

**Supplementary Table 8 Average minor allele frequency (MAF), observed heterozygosity, and inbreeding rates based on the excess of homozygosity ($F_{HOM}$) for five color-types of American mink using the whole genome sequence data.**

| Color-types | Number of animals | Average MAF | Observed heterozygosity (%) | $F_{HOM}$ |
|---|---|---|---|---|
| Demi | 32 | 0.210 | 30.54 | -0.022 |
| Pastel | 10 | 0.192 | 30.57 | -0.023 |
| Mahogany | 20 | 0.207 | 30.49 | -0.020 |
| Stardust | 7 | 0.185 | 30.53 | -0.021 |
| Black-NS [1] | 16 | 0.196 | 30.53 | -0.021 |
| CCFAR [2] | 85 | 0.211 | 30.54 | -0.022 |
| Black-ON [3] | 15 | 0.187 | 27.22 | 0.089 |
| Total | 100 | 0.198 | 30.06 | -0.005 |

[1] Black color-type from Canadian Center for Fur Animal Research (CCFAR) at Dalhousie Faculty of Agriculture (NS, Canada)

[2] All samples collected at Canadian Center for Fur Animal Research (CCFAR)

[3] Black color-type from Millbank Fur Farm (ON, Canada)

**Supplementary Table 9** The average (±SD) number of runs of homozygosity (ROH) and inbreeding rates based on ROH ($F_{ROH}$) in different color-types of American mink.

| Color-Types | Minimum Length of ROH | | | | | | | |
|---|---|---|---|---|---|---|---|---|
| | **500 kb** | | **1 Mb** | | **2 Mb** | | **4 Mb** | |
| | **Number** | $F_{ROH}$ | **Number** | $F_{ROH}$ | **Number** | $F_{ROH}$ | **Number** | $F_{ROH}$ |
| Demi | 88 ± 33 | 0.036 ± 0.014 | 29 ± 13 | 0.021 ± 0.011 | 9 ± 5 | 0.011 ± 0.007 | 2 ±2 | 0.005 ± 0.004 |
| Pastel | 87 ± 34 | 0.035 ± 0.015 | 28 ± 13 | 0.021 ± 0.010 | 8.5 ± 5 | 0.011 ± 0.007 | 2 ± 2 | 0.004 ± 0.005 |
| Mahogany | 93 ± 35 | 0.038 ± 0.015 | 30 ± 14 | 0.022 ± 0.011 | 9.2 ± 5 | 0.012 ± 0.007 | 2 ± 2 | 0.004 ± 0.005 |
| Stardust | 89 ± 34 | 0.037 ± 0.015 | 30 ±14 | 0.021 ± 0.011 | 8.7 ± 5 | 0.011 ± 0.007 | 2 ± 2 | 0.004 ± 0.004 |
| Black-NS | 89 ± 34 | 0.037 ± 0.016 | 27 ± 14 | 0.021 ± 0.011 | 8.7 ± 5 | 0.011 ± 0.007 | 2 ± 2 | 0.004 ± 0.005 |
| CCFAR | 88 ± 33 | 0.036 ± 0.014 | 29 ± 13 | 0.021 ± 0.010 | 8.7 ± 5 | 0.011± 0.007 | 2 ± 2 | 0.004 ± 0.004 |
| Black-ON | 190 ± 40 | 0.087 ± 0.044 | 57 ± 31 | 0.057 ± 0.043 | 23 ± 22 | 0.032 ± 0.038 | 2 ± 2 | 0.004 ± 0.005 |

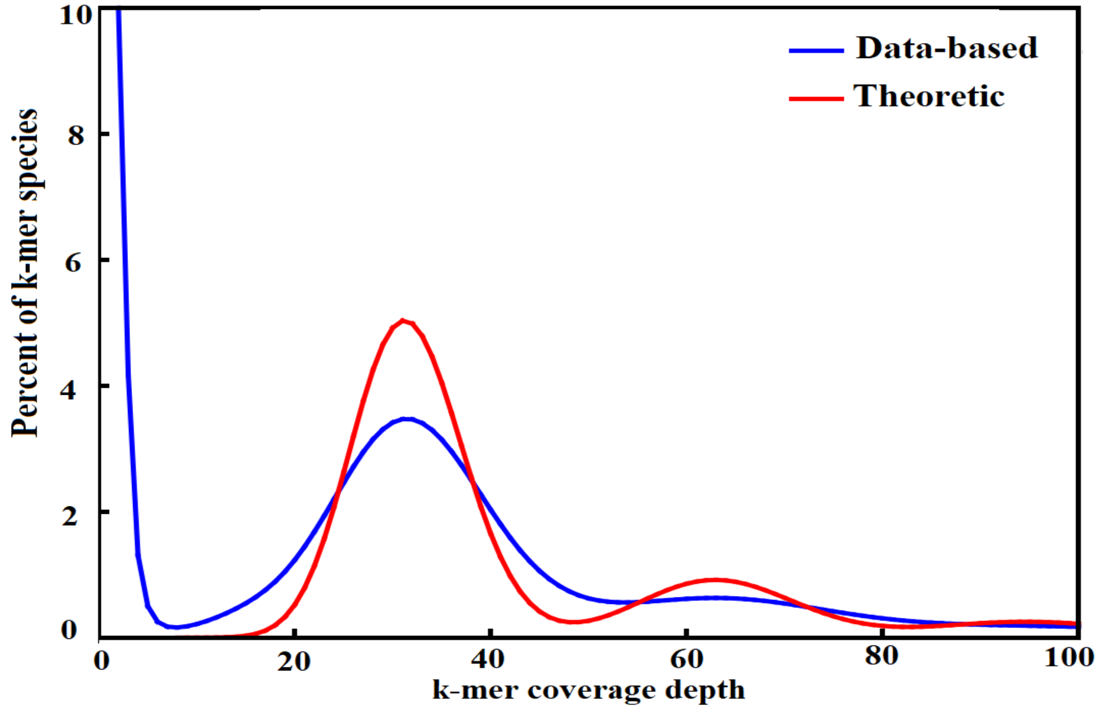


**Supplementary Figure 1 The data-based (blue) and theoretic (red) distribution of 17-mers estimated based on the BGI short reads for the American mink genome (ASM_NN_V1).**

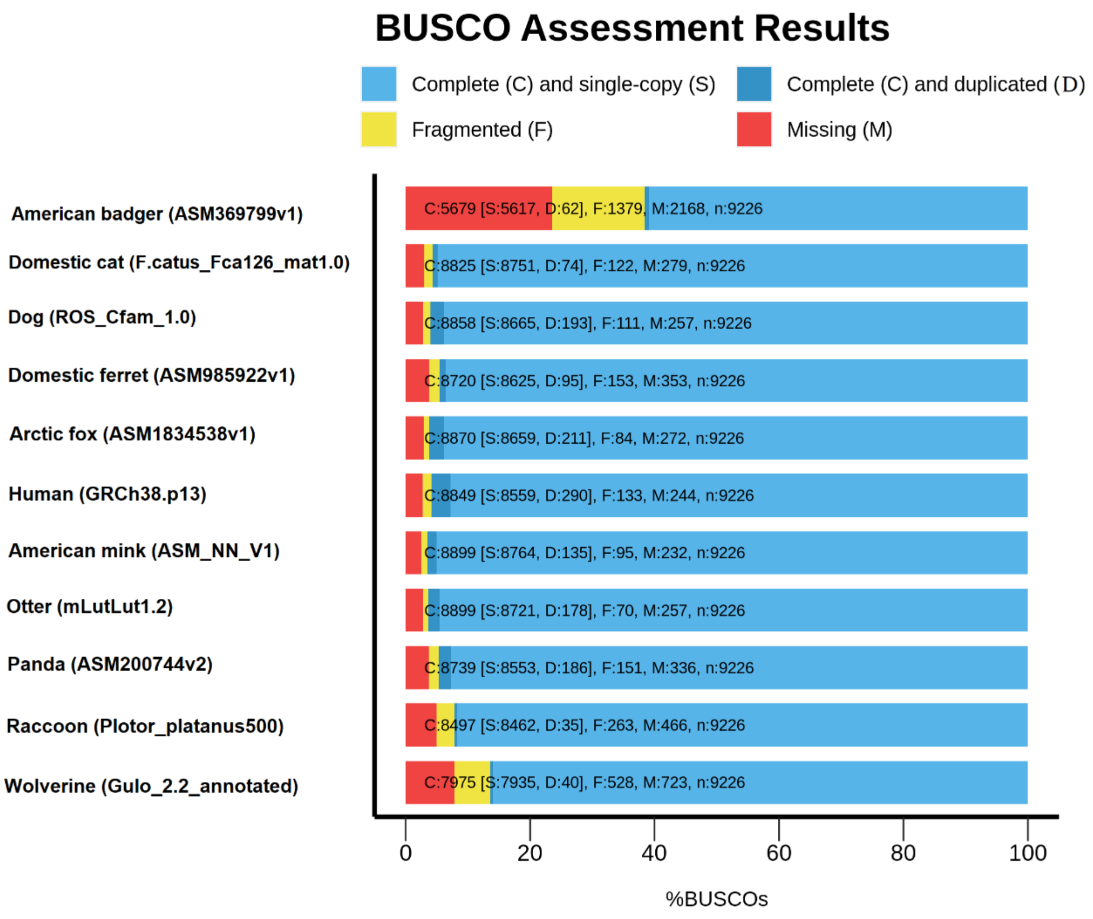


**Supplementary Figure 2 Comparison of BUSCO scores of American mink genome (ASM_NN_V1) with the genome assemblies of nine Carnivorous mammals as well as human.** The BUSCO scores were computed by BUSCO v5.2.2 using the mammalia_odb10 data set. The names of corresponding species were mentioned along with the assembly version (inside the parenthesis) applied to perform the analysis.

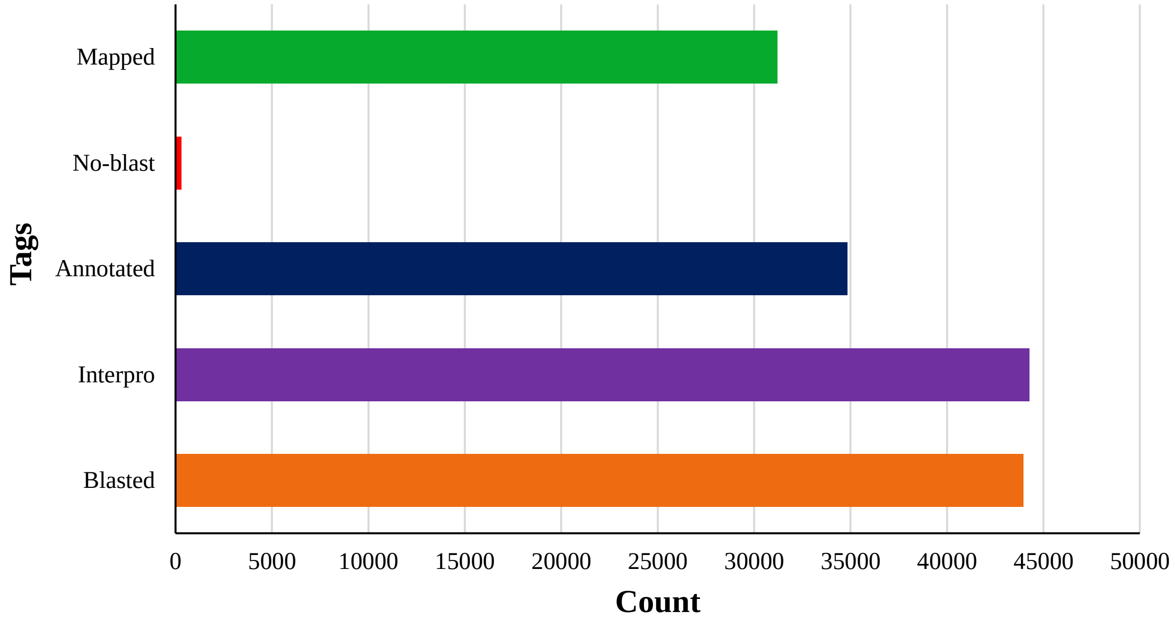


**Supplementary Figure 3 The number of American mink protein sequences retrieved by UniProtKB/Swiss-Prot Blast (orange), InterPro collection databases (purple), assigned GO terms (blue), no blasted hits (red), and those mapped to gene ontology database (green).**

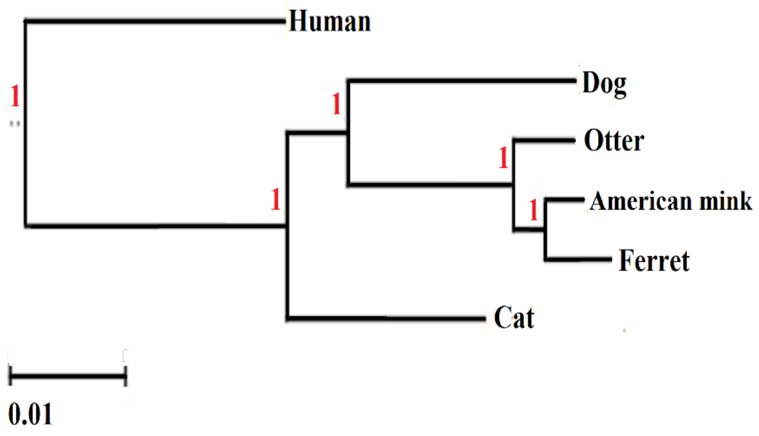


**Supplementary Figure 4 The phylogenetic tree inferred based on a concatenated alignment of 2,971 single-copy orthogroups of American mink, ferret, otter, dog, cat, and human.**

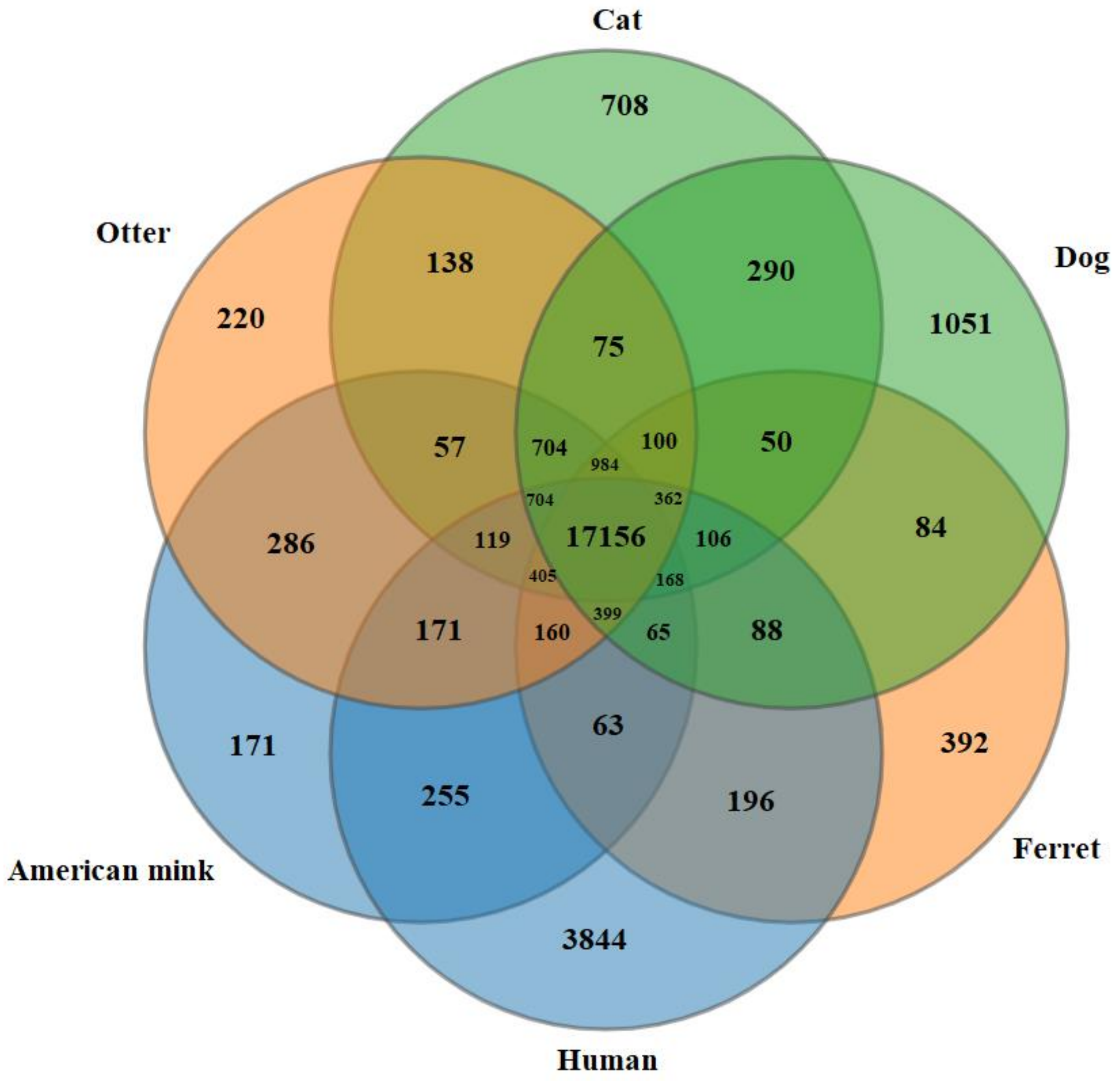


**Supplementary Figure 5 The Venn diagrams presenting the distribution of hierarchical orthogroups (HOGs) among the studied species.**

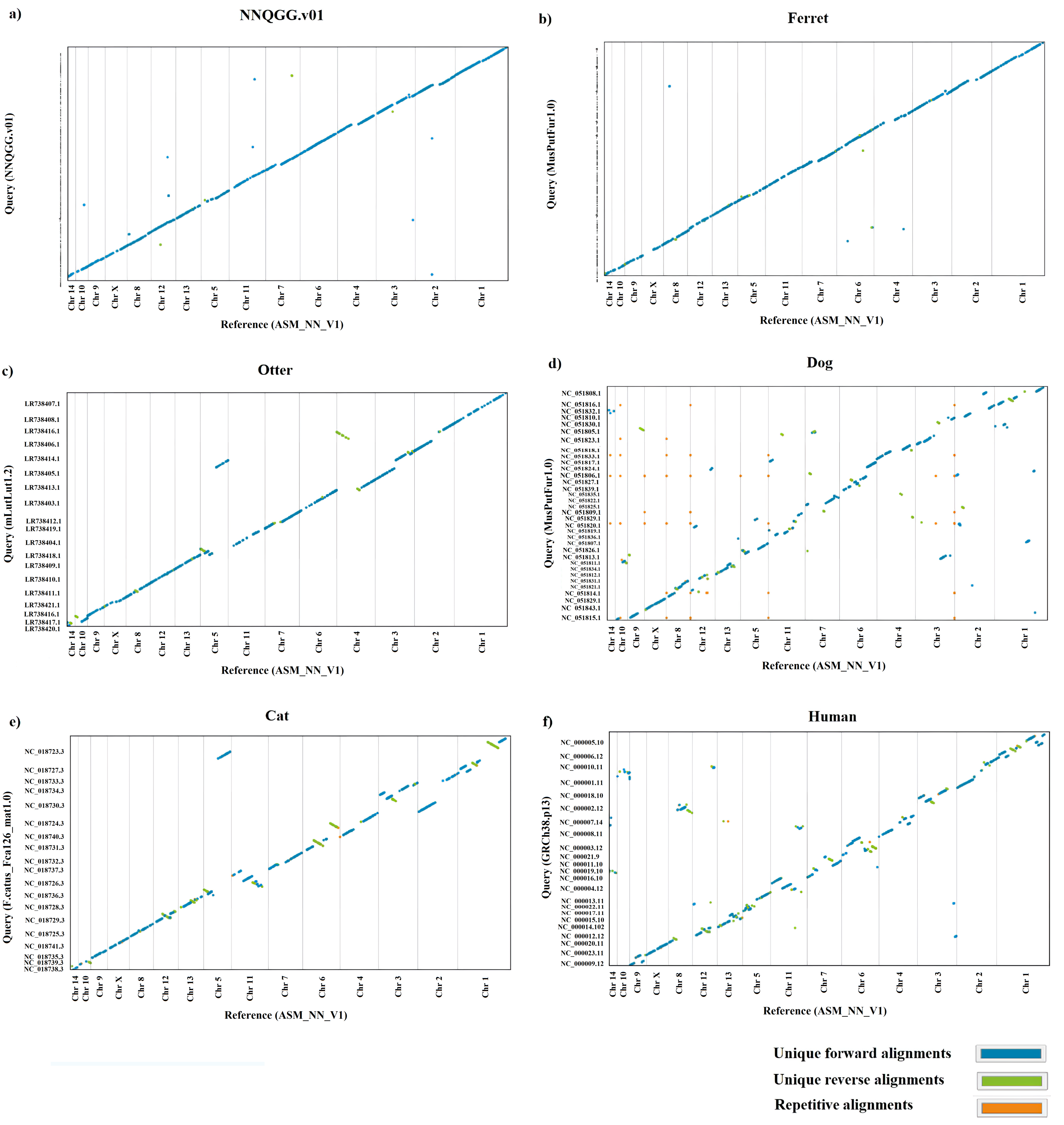


**Supplementary Figure 6 Dot plots representing the synteny and rearrangement**. Synteny blocks were presented between American mink genome (ASM_NN_V1) and **a)** first draft of mink genome (NNQGG.v01) **b)** ferret (MusPutFur1.0) **c)** otter (mLutLut1.2) **d)** dog (ROS_Cfam_1.0) **e)** cat (F.catus_Fca126_mat1.0) and **f)** human (GRCh38.p13). Since the NNQGG.v01 and MusPutFur1.0 were scaffold-scale assemblies, the chromosomes' names were not presented for them.

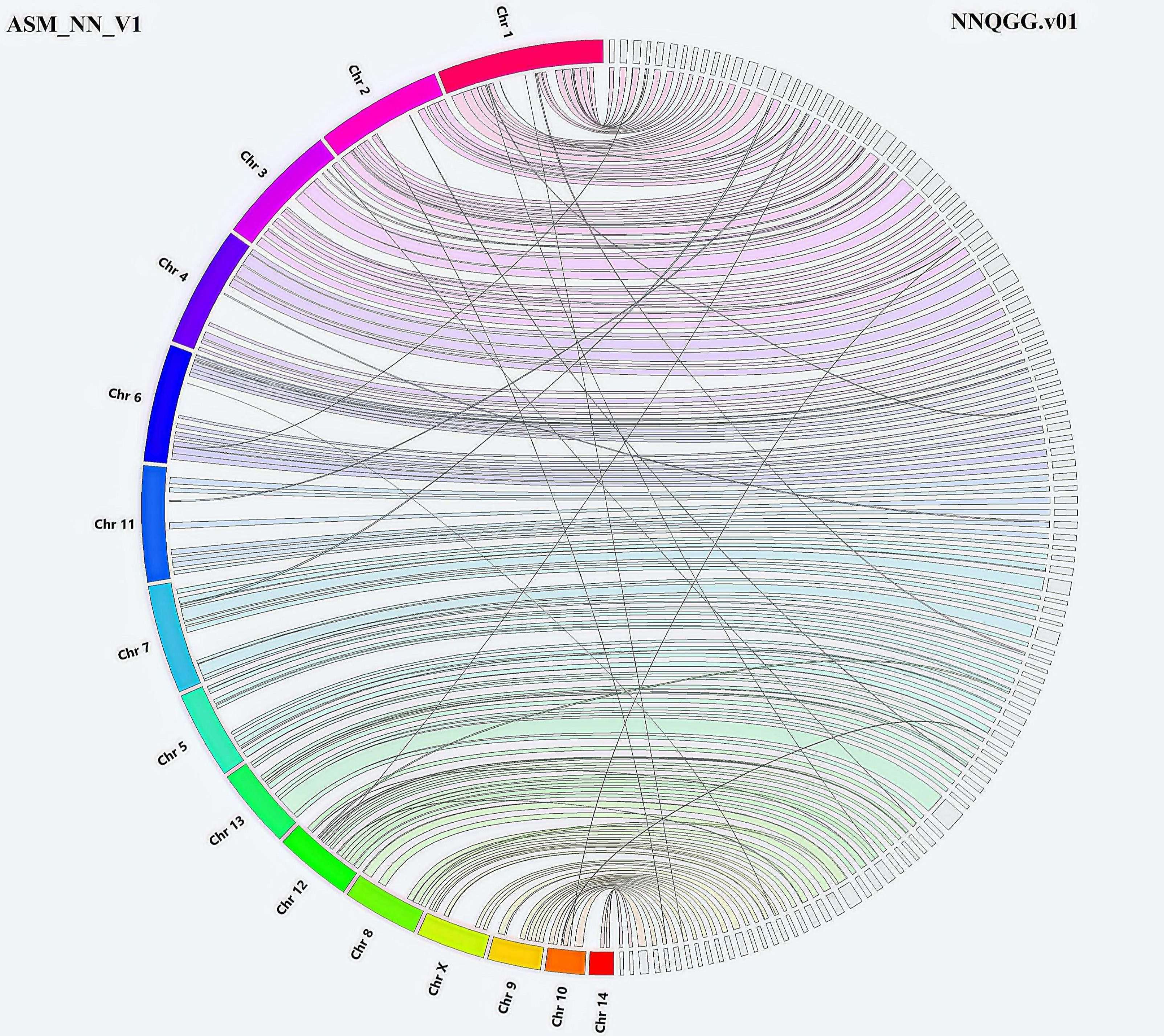


**Supplementary Figure 7 Circos plot of alignments between two American mink assemblies including ASM_NN_V1 (left) and NNQGG.v01(right).** Colors represent different chromosomes of American mink genome available form ASM_NN_V1.

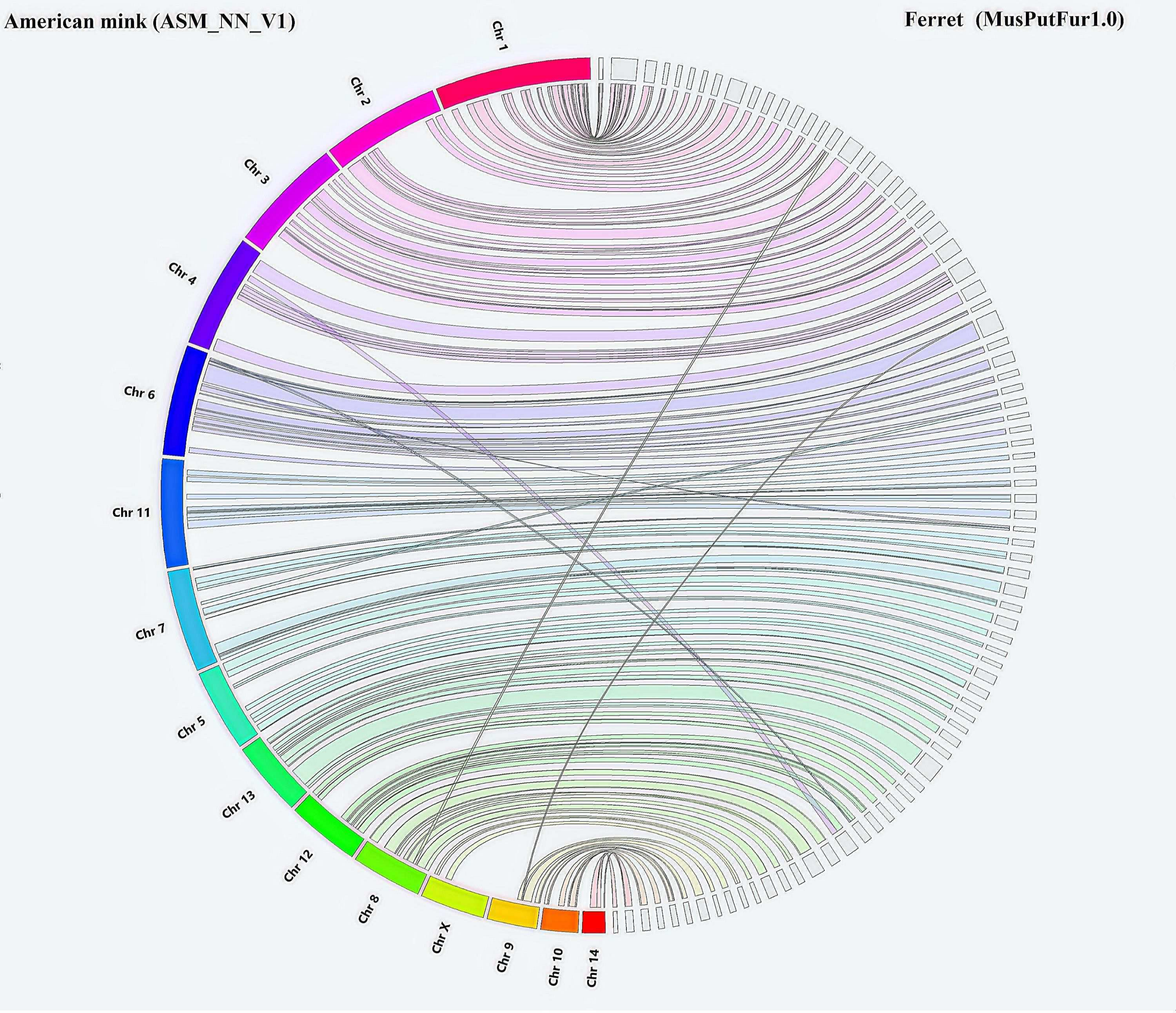


**Supplementary Figure 8 Circos plot of alignments between ASM_NN_V1 (left) and ferret genome (right).** Colors represent different chromosomes of American mink genome.

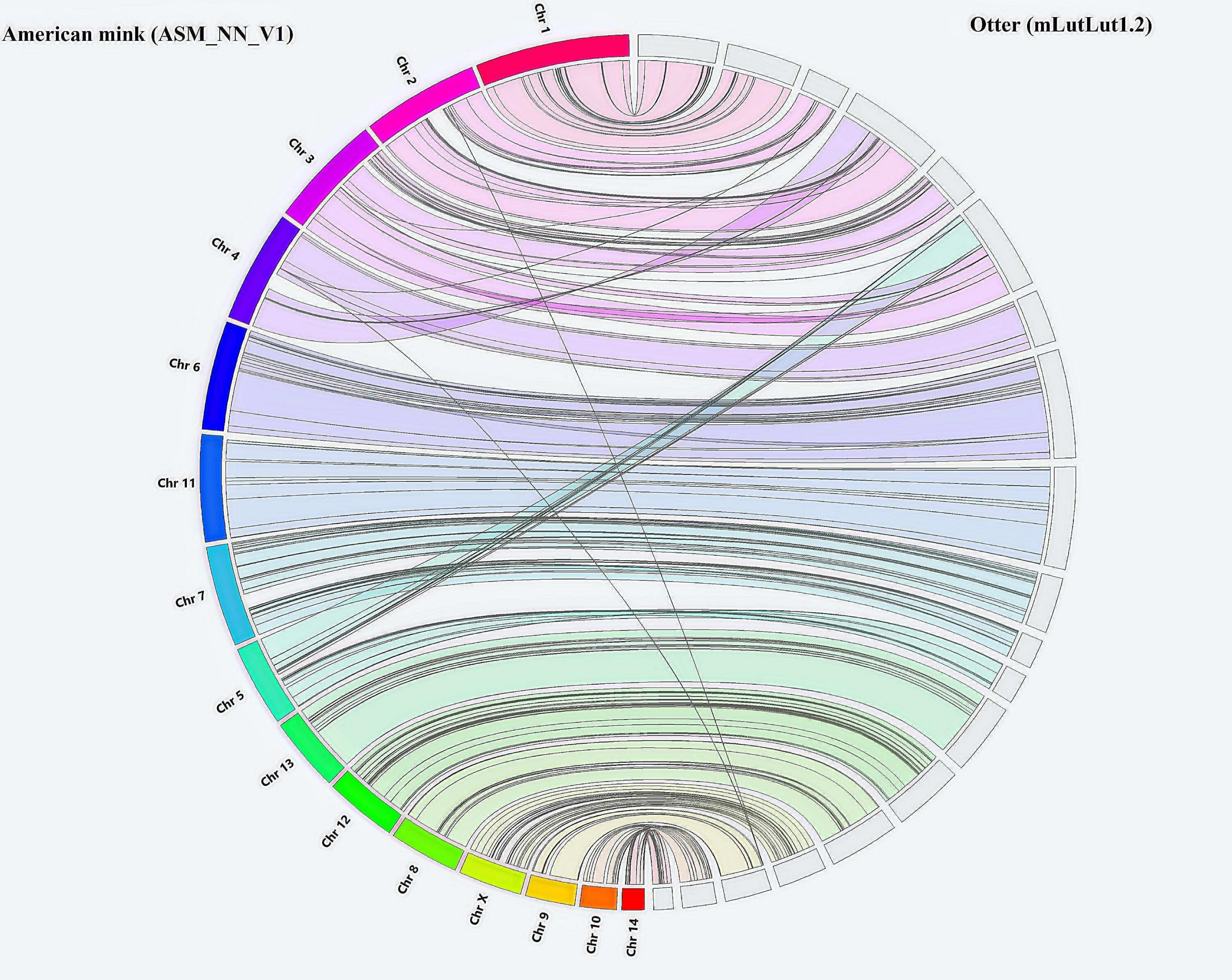


**Supplementary Figure 9 Circos plot of alignments between ASM_NN_V1 (left) and otter genome (right).** Colors represent different chromosomes of American mink genome.

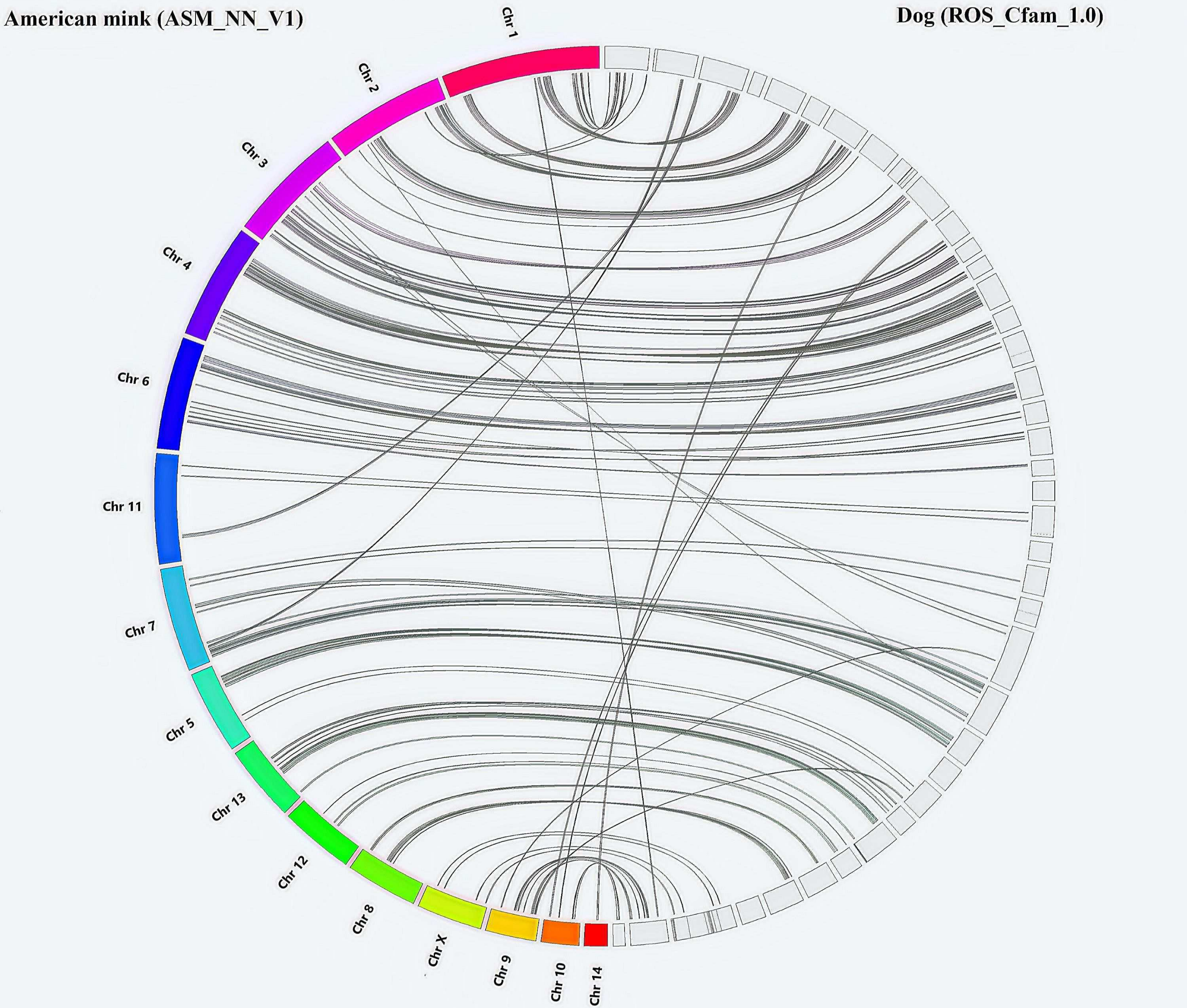


**Supplementary Figure 10 Circos plot of alignments between ASM_NN_V1 (left) and dog genome (right).** Colors represent different chromosomes of American mink genome.

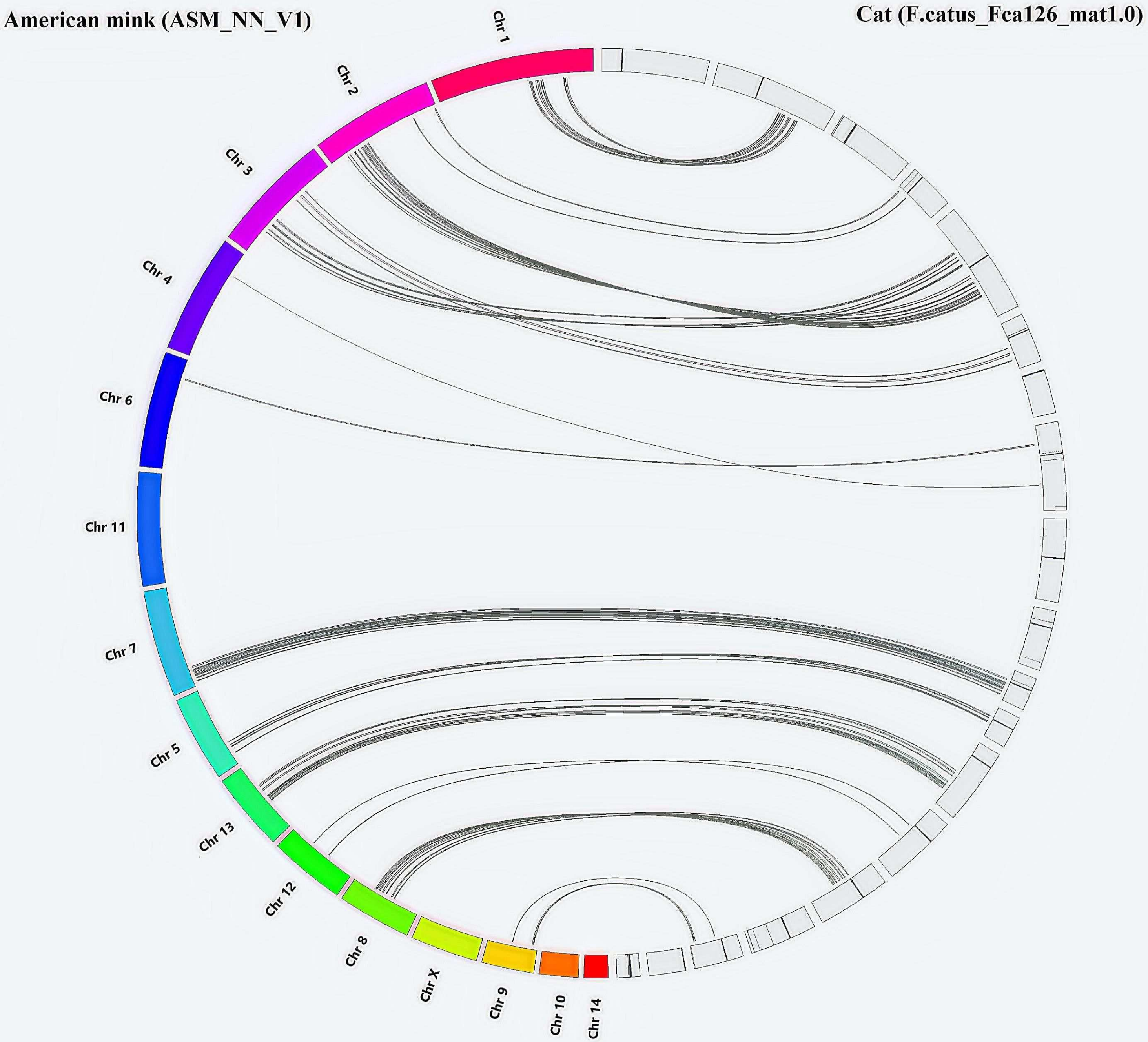


**Supplementary Figure 11 Circos plot of alignments between ASM_NN_V1 (left) and cat genome (right).** Colors represent different chromosomes of American mink genome.

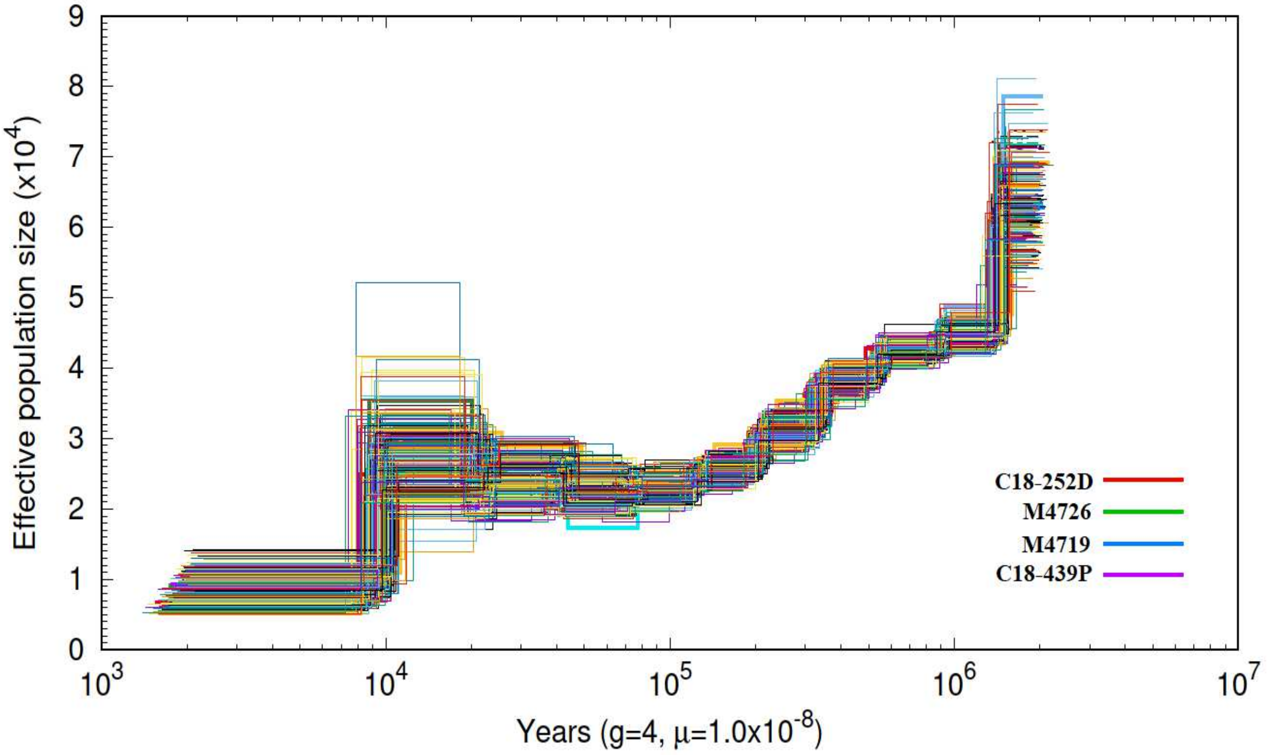


**Supplementary Figure 12 Demographic history of American mink as inferred by PSMC with 100 bootstraps per sample.** The ASM_NN_V1 was used as the reference genome with the generation time (g) of four years and mutation rate (μ) of $1\times10^{-8}$. The M4719 was the black mink used to construct the genome assembly. The M4726 was the black mink sampled from the Millbank Fur Farm and the C18-252D and C18-439P were the black and pastel mink collected from the Canadian Centre for Fur Animal Research (CCFAR) at the Dalhousie Faculty of Agriculture (Truro, NS, Canada), respectively.
