# Supplementary material for: A chromosome-level genome assembly reveals genomic characteristics of the American mink (Neogale vison)
Source: Commun Biol. 2022 Dec 16;5:1381. doi: 10.1038/s42003-022-04341-5 (PMC9757699; doi:10.1038/s42003-022-04341-5)
Supplement: Supplementary file 2 — Description of Additional Supplementary Data [file 42003_2022_4341_MOESM2_ESM.docx]

**Description of Additional Supplementary Files**

**File name:** Supplementary Data 1

**Description:** The source data behind Fig. 3. The major GO classes are included in all levels of molecular function GO terms.

**File name:** Supplementary Data 2

**Description:** The upgrade of genomic locations for the genes identified for mink traits in the previous publications.

**File name:** Supplementary Data 3

**Description:** The lists of aligned regions between ASM_NN_V1 and other studied genomes.
